# Correcting for case-mix shift when developing clinical prediction models

**DOI:** 10.1186/s12874-025-02621-2

**Published:** 2025-08-01

**Authors:** Haya Elayan, Matthew Sperrin, Glen P. Martin, Niels Peek, Frieder Braunschweig, Jonas Faxén, Joakim Alfredsson, David A. Jenkins

**Affiliations:** 1 Division of Informatics, Imaging and Data Science, Faculty of Biology, Medicine and Health, University of Manchester, Manchester, UK; 2 Department of Cardiology, Karolinska University Hospital, Stockholm, Sweden; 3 Department of Physiology and Pharmacology, Karolinska Institute, Stockholm, Sweden; 4 Department of Health, Medicine and Caring Sciences, Linköping University, Linköping, Sweden; 5 NIHR Manchester Biomedical Research Centre, Manchester University NHS Foundation Trust, Manchester Academic Health Science Centre, Manchester, UK

**Keywords:** Clinical Prediction Models, Membership Propensity Score, Weighted Logistic Regression, Case-Mix Shift

## Abstract

**Background:**

When developing a clinical prediction model (CPM), a case-mix shift could occur in the development dataset where the distribution of individual predictors changes, potentially affecting model performance. This study exploits the case-mix shift that is already observed in the development dataset to address the case-mix shift between the development and deployment phase of a CPM.

**Methods:**

We propose a Membership-based method to correct for case-mix shift in the development phase of CPMs. This method uses a probabilistic similarity metric to re-weight data samples in the source set (before the case-mix shift) to more closely match the target set (after the case-mix shift), assuming the target set reflects the target population. We apply the proposed method in a real-world dataset of myocardial infarction patients with out-of-hospital cardiac arrest within 90 days as the outcome. We design nine scenarios (including case-mix shift and no case-mix shift with a range of target/source sets sample sizes) to explore the impact on predictive performance of CPM developed with the proposed method in comparison to CPMs developed by either using all data samples but ignore the shift, or only using the most recent data. We report calibration and discrimination on development and 200 bootstrap samples.

**Results and Conclusions:**

The proposed method shows promise in accounting for case-mix shift when developing a CPM, particularly when the target set sample size is insufficient. In a partial case-mix shift scenario with an insufficient target sample size, the Membership-based model achieved an optimism-adjusted calibration slope (c-slope) of 0.98, outperforming other models. Conversely, when the target set sample size is sufficient, the Unweighted model on target data only had an optimism-adjusted c-slope of 0.95, compared to 0.92 for the Membership-based model. In complete case-mix shift cases, the Membership-based and Unweighted on target data only models performed similarly. Both achieved an optimism-adjusted c-slope of 0.77 with insufficient target sample size, and optimism-adjusted c-slope of 0.94 with a sufficient target sample size. Further investigation and testing are needed, as well as accounting for other types of data distribution shift to improve model fit for the latest distribution shift in the development dataset.

**Supplementary Information:**

The online version contains supplementary material available at 10.1186/s12874-025-02621-2.

## Background

Clinical prediction models (CPMs) are models/algorithms that compute the risk of a diagnostic or prognostic outcome, given a set of characteristics (predictors) related to a patient, disease, or treatment [1]. CPMs have become an important tool in several phases of clinical pathways to improve health services and quality of care. For example, EuroSCORE estimates the risk of mortality after cardiac surgery and is used as a benchmark for assessing the quality of cardiac surgical services [2]. QRISK3 calculates the risk of developing ischemic heart disease or ischemic cerebrovascular disease over the next 10 years for a person and is used for deciding on primary prevention interventions [3].

The healthcare environment is dynamic. Demographics of population, prevalence of diseases, clinical practices and health policies may change over time or space, and such changes could happen gradually, abruptly, incrementally, or reoccur after some time (seasonality). Recently, changes in care delivery during the COVID-19 pandemic highlight how quickly and critically the environment in which models operate can shift given changes in data collection, patient case mix, and clinical decision making [4]. Such changes lead to a phenomenon in the observed health data, referred to as Data Distribution Shift where differences arise over time or over space in the underlying distribution of the data [5, 6].

In the context of predictive modelling, data distribution shift could occur if the development dataset is collected over a time period in a non-stationary environment, causing the distribution to change as time passes. Alternatively, it could occur if the dataset is collected from multiple settings or healthcare centres where there is heterogeneity between the settings/centres’ data [7, 8]. Among the data distribution shift types, Case-mix shift refers to changes observed in the distribution of individual predictors, *P*(*X*), of the data but the conditional probability of the outcome given a predictor remains the same *P*(*Y*|*X*). The case-mix may change as patients age, new established healthcare facilities bring new patients into the system with new or different demographics, the data of the patients is collected from different clinical settings, or the model is transferred to a different clinical setting [9–13]. For example, when a model developed on data from adults is used to make predictions on data of children, a case-mix shift may occur as the characteristics of the two age groups differ [8, 14, 15].

The ways in which CPMs are usually developed either ignore distributional shifts by using all available data samples with equal weights, or the CPM is developed using only data from a specific time period (or geographical location) that is more reflective of the data in which the model will be implemented within[16–20]. The first means that the model does not capture the distributional changes that are present in the data, and the second wastes information and reduces the required sample size for building the model, making it prone to overfitting [21–25]. In both situations, the predictive performance may be negatively affected [5, 8, 26]. This is particularly problematic if the CPM will be used for patient-level clinical decision-making, which requires highly accurate models that consistently perform well [27].

While a CPM models the probability of the outcome given the input *P*(*Y*|*X*), the performance of the model should not be directly influenced by the nature of the case-mix shift. This is true when we are estimating *P*(*Y*|*X*) using infinite samples with some degree of overlap, as asymptotically the correct model will be recovered. However, when estimating *P*(*Y*|*X*) with a finite sample, it is preferable for our estimate to be more accurate in places where there will be most data at deployment phase rather than in development phase, where the CPM tends to automatically do the latter. As we are primarily interested in the deployment dataset, there are often aspects of case-mix shift that can be observed already in the development dataset. This case-mix shift in the development dataset could be exploited to address the case-mix shift between the development and deployment phase.

In this study we aim to develop a method that can be used to improve the prediction of CPMs in presence of case-mix shift in the development dataset and demonstrate the use of this method under a set of scenarios using real-world data. We consider that the latest distribution shift in the population used to develop the model reflects the distribution of the data that the model will be implemented within - the target population [18, 21, 28]. Therefore, this study is a phase one and two of methodological research study [29]. Specifically, throughout this paper, we assume that there is a change in the distribution of individual predictors, *P*(*X*), within the data used to develop the CPM, before or after a given time point (we use time for conceptual ease, but the concepts apply equally to different geographical locations). Our proposed method accounts for case-mix shift by adapting the membership model concept for re-weighting the data samples, to correct for distributional changes. We apply the proposed method in a real world dataset and compare the predictive performance of the proposed method, where the calibration and discrimination will be calculated, with two existing CPM development methods.

The remainder of this study is as follows: Methods section proposes the Membership-based method for re-weighting the data samples to account for the case-mix shift. Case study section discusses the applied case study including the dataset used, the selected predictors, the develop models, the study design, and themes and scenarios conducted to compare the predictive performance of the proposed method with two existing CPM developing methods. Results section presents the collected results. Finally, Discussion section discusses the results, reports the main findings, the study strength and limitations, and the study recommendations.

## Methods

Let *X* denote a $N \times K$ matrix of predictor variables, and *Y* denote a vector of length *N* of outcomes from a joint distribution *P*(*X*, *Y*). Assume we aim to use these data to develop a model to estimate *P*(*Y*|*X*) and a case-mix shift has occurred in one component of the joint distribution of the data from the population used to develop the model *P*(*X*, *Y*). Specifically, the probability density of the input *P*(*X*) changes after a given time-point, but the conditional probability of the output given the input *P*(*Y*|*X*) remains the same.

Let *R* denote an indicator for the data being from a certain distribution, where $R = 0$ indicates the data being from the distribution after the latest case-mix shift $P(X, Y| R=0)$, and $R = 1$ indicates the data being from the distribution before the latest case-mix shift $P(X, Y| R=1)$. Additionally, we assume that the latest distribution shift $P(X, Y| R=0)$ reflects the distribution of the data from where the model will be implemented - i.e. the target population. The case-shift means that

1 $$\begin{aligned} P(X, R=1) & \ne P(X, R=0)\nonumber \\ P(Y|X, R=1) & = P(Y|X, R=0) \end{aligned}$$

We refer to the subset of $P(X,Y| R=1)$, before the latest distribution shift, as ‘source’ data, and the subset of $P(X,Y| R=0)$, after the latest distribution shift, as ‘target’ data. The source data, denoted as $source\_set$, is of size *s* (where $s < N$), and the target data, denoted as $target\_set$, is of size *n* (where $N=s+n$). We propose to re-weight data in such a way as to ‘correct for’ case-mix shift based on a membership (probabilistic) model. The proposed approach will produce a set of weights that will be used to construct a weighted source set from $source\_set$, by giving individuals from $source\_set$ high weights when they are relevant to $target\_set$ and low weights when they are less relevant to $target\_set$.

### Membership-based weights

We propose the use of propensity score methodology to construct weights, emphasizing its application in predictive modeling, while explicitly precluding its use for the estimation of causal effects. We attempt to weight based on importance, such that we transform the distribution of the source set to closer represent the target set using inverse-odds weights obtained from estimating the propensity score [30].

We first use the membership propensity score *PS*, defined as the conditional probability of an individual *i* being a member of the $source\_set$, given its observed values from a set of predictor variables $\varvec{X}_i =(X_{i1},...,X_{iK})$, where *K* denotes the number of predictor variables, as follows:

2 $$\begin{aligned} PS_{i}= P(R= 1| \textbf{X}_{i}) \; for \; i \in \{1,...,s\} \end{aligned}$$

A binary logistic regression model (membership model) can be used to estimate the *PS*, where the outcome of the membership model is 0 for individuals of the $target\_set$ and 1 for individuals of the $source\_set$ [31]:

3 $$\begin{aligned} PS_{i} = \frac{1}{(1 + e^{-(\alpha + \sum \nolimits _{k=1}^K \beta _kX_{ik})})} \; for \; i \in \{1,...,N\} \end{aligned}$$

Finally, to produce a weight $w_i$ for an individual *i* from the $source\_set$, the Membership propensity score of $target\_set$ is divided by the Membership propensity score of $source\_set$, multiplied by the sample size ratio where the sample size of $source\_set$ divided by the sample size of $target\_set$, as follows:

4 $$\begin{aligned} w_i & = min \left( \frac{P(R= 0| \textbf{X}_{i}) }{P(R= 1| \textbf{X}_{i}) } \times \frac{s}{n}\,\ 1 \right) \nonumber \\ & = min \left( \frac{ 1- (P(R= 1| \textbf{X}_{i}))}{P(R= 1| \textbf{X}_{i})} \times \frac{s}{n}\,\ 1 \right) \end{aligned}$$

This takes into account giving the less relevant individual lower weight when has lower probability of being member of $target\_set$. As well as giving the individual a higher weight (up to 1) when has higher probability of being member of $target\_set$. Limiting the weights up to 1 prevents the potential for generating overoptimistic standard errors within the prediction model that will be developed on the weighted dataset. This precaution is necessary because oversampling the more relevant individuals would artificially inflate the effective sample size.

Alternatively, a scaling factor *c* can be applied to ensure that the effective sample size of the source set equals the actual sample size of the source set, when the former is larger than the latter. Consequently, the total effective sample size will be equal to the actual total effective sample size, as follows:

5 $$\begin{aligned} w_i = \frac{1- (P(R= 1| \textbf{X}_{i}))}{P(R= 1| \textbf{X}_{i})} * \text {c} \end{aligned}$$

where:

$$\begin{aligned} \text {c} = \frac{s}{\sum \nolimits _{i=1}^s w_i} \end{aligned}$$

### Developing a weighted model

The proposed method can be used for different types of outcomes and with different types of models. By utilizing logistic regression as a specific example, our proposed method uses a weighted regression model, with *V* set of predictors where $V \le K$, to weight each individual from the $source\_set$ in the log likelihood based on their relatedness to the target set $target\_set$, while weighting individuals from the target set with weight equal to 1. Our proposed method can be extended to weighted linear regression or weighted survival models. Prior research [32–38] has introduced other weighting schemes applied in similar contexts.

Let individual $i \in (1,...,N)$ and $w_i$ is the corresponding individual weight. The weights are then included in the log-likelihood (*LL*) as follows:

6 $$\begin{aligned} \text {LL} = \sum \limits _{i=1}^N (y_i*\text {log}(p_i) + (1-y_i)*\text {log}(1-p_i)) * w_i \end{aligned}$$

where *p* is the predicted probability for a binary outcome, and *y* is an observed outcome.

## Case study

To illustrate the above weighting scheme, we developed a weighted model and compared its predictive performance with models developed by either ignoring case-mix shift or only developed on most recent data in a real-world dataset. We varied the combination of the development dataset to source and target sets to achieve different uses, as well as, varied the proportions of the source or target sets to design different scenarios for each use. We describe the dataset, study design, and themes and scenarios in this section.

### Dataset

The Swedish Web-System for Enhancement and Development of Evidence-Based Care in Heart Disease Evaluated According to Recommended Therapies (SWEDEHEART) is a national quality registry including patients admitted to a coronary care unit or other specialized facility with a suspected or definite acute coronary syndrome (ACS). Collected data include demographics, medical history, baseline variables, medical treatment, procedures, and complications during the hospital course, as well as medication at discharge [39]. In the current study, cross-linked data from SWEDEHEART, the Swedish Cardiopulmonary Resuscitation Registry, and the Swedish Pacemaker and Implantable Cardioverter-Defibrillator (ICD) Registry were used. Patients, registered in SWEDEHEART between 2009 and 2017, with myocardial infarction (MI), who had undergone coronary angiography and were discharged alive without a prior ICD were included [40].

The outcome is out-of-hospital cardiac arrest (OHCA) within 90 days after discharge from coronary care for ACS. Among 166,394 observations, 351 (0.21%) experienced OHCA within 90 days. A total of seven variables; Age, Sex, Diabetes, Atrial fibrillation/flutter (AF), Body mass index (BMI), Left ventricular ejection fraction (LVEF), and Estimated glomerular filtration rate (eGFR) were selected for inclusion as predictor variables to predict the OHCA with 90 days, based on previously published model by Faxén et al. [40]. For illustration, the same set of variables is used to calculate the weights for the proposed method and as predictor variables in the prediction models. Table 1 describes the list of used predictors. For the purpose of this study we used a cut-off at 40% to define patients with a reduced LVEF (<40%) or preserved LVEF ($\ge$ 40%). To account for data missingness, a single imputation method (Predictive Mean Matching) is used for missing data imputation with including the outcome. Furthermore, the minimum sample size criteria was calculated using the method of Riley et al. [22], where we assumed an event prevalence of 0.0021, a target shrinkage factor of 0.9, number of predictors of 7, and c-statistic of 0.75 [40], this gave a required sample size of 33,123. This minimum sample size criteria was met during the models’ development.

**Table 1:** List of SWEDEHEART variables used as predictor variables

Classification	Variable name	Units, categories	Description
Demographics	Age	Years	Age
Demographics	Sex	Male, Female	Sex
Clinical characteristics	BMI	kg/m2	Body mass index
Medical history	Diabetes	No, Yes	Diabetes
Circulating biomarkers	eGFR	mL/min/1.73m2	Estimated glomerular filtration rate (^*^CKD-EPI)
ECHO measurements	LVEF	below 40%, above 40%	Left ventricular ejection fraction (echocardiography)
Medical history	Atrial fibrillation/flutter (AF)	No, Yes	Atrial fibrillation or atrial flutter

^*^CKD-EPI = Chronic Kidney Disease Epidemiology Collaboration

### Study design

The primary aim of this case study is to compare the predictive performance of the model developed using the proposed method with two existing model development methods. The development dataset was divided into source and target sets based on different uses and various proportions of the source or target sets to design different scenarios.

The models developed were as follows:

1. Unweighted model on all data: A logistic model trained on the all development dataset samples.
2. Unweighted model on target data: A logistic model trained on the target set only to account for the most recent change.
3. Membership-based model on all data: A weighted logistic model with Membership-based weighting trained on all data while re-weighting the source data samples using the propensity score weighting procedure and weighting the target data samples as 1.

Within the dataset, there were changes in the event proportion through time (event rate shift) in addition to case mix shift. Given that our proposed method only addresses the case-mix shift, we developed additional models to account for the shift in the outcome. The calibration in the large (CITL) of each new model was adjusted through adding a dummy variable for the target set membership *R* [41]. The dummy variable *R* indicates the absence or presence of the effect of target dataset membership that causes a shift for the outcome, where it takes a value of 0 if the samples belong to the target dataset, and a value of 1 if the samples belong to the source dataset. This dummy variable was added as an additional predictor in the new developed models that utilize all the available data samples.

Both apparent and bootstrap internal validation results of predictive performance (CITL, calibration slope, discrimination, and Brier score) are reported from the target set. For calibration, the calibration in the large was obtained by estimating the intercept $\alpha$ through fitting each developed model to the outcomes with fixing the linear predictor of each model as an offset variable (the regression coefficient is constrained to be 1). Calibration slope was obtained by estimating the regression coefficient $\beta$ through fitting each developed model to the outcomes and using the linear predictor of each model as the only covariate. For discrimination, the concordance statistic C was used, which is equivalent to the area under the receiver operating characteristic (ROC) curve. The area under the precision and recall curve (AUC-PR) was also used to calculate the area under the precision and recall curve, rather than the sensitivity and specificity curve, as it provides better agreement with positive predictive value at low outcome prevalence. Finally, as a measure of an overall performance, the Brier score was studied where lower values represent better performance.

The adjusted optimism for each performance metric was estimated to provide an estimate of predictive performance after removing in-sample optimism. Developing a model in a dataset and then testing the model in that same data (without adjustment) will lead to overly optimistic performance estimates [26, 42]. The adjusted optimism for each performance metric was estimated by calculating the difference between the bootstrap apparent metric and bootstrap test metric of the 200 bootstrap samples, then subtract the mean optimism over 200 bootstrap samples from the development apparent metric. Please refer to the supplementary materials for illustration of estimating the optimism-adjusted performance metric [see Additional file 1]. The R code for Membership-based method is available on GitHub (https://github.com/HayaElayan/Accounting-for-Case-Mix-Shift-in-CPM-Development).

### Effective sample size of source dataset

Two approaches were implemented to control the effective sample size of the source dataset, ensuring it remains equal to or smaller than the actual sample size. The models were reported with the following weight adjustments: 1) no control over the effective sample size (baseline propensity score), 2) weights limited to a maximum of 1, and 3) a scaling factor applied only when the effective sample size exceeded the actual sample size of the source data. Table 2 lists all the options followed for the developed models.

**Table 2:** List of modelling options

Modelling option	Unweighted model on all data	Unweighted model on target data	Membership-based model on all data
Use all data	✓	✗	✓
Use target data only	✗	✓	✗
Adjust the CITL	✓	✗	✓
Weighting (no weight limit)	✗	✗	✓
Weighting (weight up to 1)	✗	✗	✓
Weighting (scaled weight) if required	✗	✗	✓

### Themes and scenarios

Four main themes were implemented to compare the performance of the developed models. These themes involved varying the combination of the data from the source and target datasets to illustrate specific uses. Within each theme, two or three similar scenarios were designed by varying the proportion (rate) of the target or source datasets.

Theme 1 (Complete case-mix shift) and theme 2 (Partial case-mix shift) introduce a case-mix shift in one continuous variable in the dataset, while maintaining the other variables in the null case without shift. Theme 3 (Insufficient Target set) tests the case when target data (alone) is not of sufficient sample size to develop the models on, with fixed sample size for the source dataset and under no case-mix shift. Finally, Theme 4 (Borrowing strength from Source set) tests the usefulness of borrowing strength from the source data at different rates with a fixed sample size for the target dataset, under no case-mix shift. In Themes 1 and 2, the split in the development dataset into source and target sets by age cut-off was inspired from the shift in the age mean in EuroSCORE prediction model [6]. This shift could also represent data from different domains, such as patients from different healthcare settings (e.g., primary versus secondary care), inpatients versus outpatients, or varying age categories (e.g., adults versus children). These domains often demonstrate differences in patient case mix, reflected in variations in the distribution and range of predictor values [7, 8]. Therefore, in Theme 1 we completely separated the age variable, where the source set consists of all individuals with the ages above 75 and the target set consists of all individuals with the ages below 75. While in Theme 2, the age variable was partially separated to have an overlap between the source set that contains ages between [18, 80], and target set that contains ages between [57, 103].

In Themes 3 and 4, the development data set was split into source and target data sets based on calendar time, where the target set represented the most recent data. The split was chosen to effectively demonstrate the methodology, rather than based on an intrinsic property of the data. In practice, the dichotomy of time used to define target and source datasets should be informed by the clinical context, such as changes in clinical guidelines, changes in clinical coding systems, changes in underling patient population. This approach can also be useful in scenarios where separate datasets are available —for instance, a large source dataset and a smaller target dataset —making the distinction more a function of data availability than population differences. Additionally, it may be relevant in situations involving shock to the system, such as COVID-19 happening suddenly, or in cases of transfer learning, where a model initially developed for one condition, such as pneumonia, is adapted for another, such as COVID-19, as frequently occurred during the early stages of the pandemic [43]. We also considered the calculated minimum required sample size for the development dataset to define the target set in some scenarios. Also in practice, the certainty of the presence or absence of case-mix shift never exists; the absence of evidence of case-mix shift does not necessarily imply there is no shift. Therefore, we have included Themes 3 and 4 to explore the properties of the method under the ‘no case-mix shift’ scenario. It is important to ensure the applicability of this method both in the absence and presence of shift; as it is difficult to detect the shift practice. Modelers may opt to use this method regardless of whether a shift is identified, thus requiring us to demonstrate the properties of the method in a ‘no case mix shift’ situation.

Each scenario’s name corresponds to the use of the theme it belongs to, and the scenarios within the same theme are differentiated based on the proportion of the target or source datasets. For example, the use of theme 2 is accounting for the case-mix shift with partial separation, in scenario 3 the proportion of the target dataset is lower than that in scenario 4, thus, “Low Target” is added to scenario 3 name which becomes “Partial Case-Mix/Low Target”, while “High Target” is added to scenario 4 name which becomes “Partial Case-Mix/High Target”. Table 3 shows the scenarios’ details for theme 1, 2, and 3, as well as, source and target sets selection. Please refer to the supplementary materials for further details on theme 4 and scenario 7 from theme 3 [see Additional file 1].

**Table 3:** Themes and scenarios details

Theme	Scenario	Source set selection	Target set selection	Total sample size	Is Target set of sufficient sample size?	Target set proportion
Theme 1: Complete case-mix shift	1) Complete Case-Mix/Low Target	Sample of size 92734 of all individuals with ages between [18, 80]	random sample of size 20000 of individuals with ages between [57, 103]	112734	Not sufficient	18%(low)
Theme 1: Complete case-mix shift	2) Complete Case-Mix/High Target	Sample of size 92734 of all individuals with ages between [18, 80]	random sample of size 73660 of individuals with ages between [57, 103]	166394	Sufficient	44%(high)
Theme 2: Partial case-mix shift	3) Partial Case-Mix/High Target	Sample of size 98716 of all individuals with ages >75	random sample of size 67678 of individuals with ages <= 75	166394	Sufficient	41%(high)
Theme 2: Partial case-mix shift	4) Partial Case-Mix/Low Target	Sample of size 98716 of all individuals with ages >75	random sample of size 20000 of individuals with ages <= 75	118716	Not sufficient	17%(low)
Theme 3: Insufficient Target set (no case-mix shift)	5) Insufficient Target/Low Target	Sample of size 70269 of all individuals from years 2006 to 2010	random sample of size 5000 of individuals from year 2017	75269	Not sufficient	7% (low)
Theme 3: Insufficient Target set (no case-mix shift)	6) Insufficient Target/Medium Target	Sample of size 70269 of all individuals from years 2006 to 2011	random sample of size 11065 of individuals from years 2016 and 2017	81334	Not sufficient	14% (medium)

## Results

### Effective sample size

As shown in Table 4, the effective sample size of the proposed Membership-based method with the limiting the weight up to 1 was smaller compared to the baseline propensity score method in scenarios 3 Partial Case-Mix/Low Target and 4 Partial Case-Mix/High Target. Moreover in scenarios 1 Complete Case-Mix/High Target and 2 Complete Case-Mix/Low Target under the case-mix shift with complete separation, the sum of weights of the source set for the Membership-based model was near zero, as propensity score strictly did not allow for extrapolation, which made the effective sample size in these two scenarios approximately the same as the effective sample size for the Unweighted model on target data. Also, scaling the weights approach was not implemented for the Membership-based method because the effective sample size was less than the actual sample size of the source dataset in all scenarios.

**Table 4:** Weights and total effective sample size for models

Scenario	Scenario 1	Scenario 2	Scenario 3	Scenario 4	Scenario 5	Scenario 6
Membership-based source set weights (no weight limit)	0	0	73772	72129	70272	70271
Membership based model effective sample size (no weight limit)	67678	20000	93772	145789	75272	81336
Membership based source set weights (weight up to 1)	0	0	44283	47637	64571	64777
Membership based model effective sample size (weight up to 1)	67678	20000	64283	121297	69571	75842
Unweighted model on all data effective sample size	166394	118716	112734	166394	75269	81334
Unweighted model on target data effective sample size	67678	20000	20000	73660	5000	11065

Limiting the weights up to 1 or scaling the weights with scaling factor were implemented to prevent generating overoptimistic standard errors within the prediction model developed with the proposed membership weighted method. Please refer to the supplementary materials for the standard errors of all models’ coefficients across all scenarios and the effective sample size for rest of scenarios [see Additional file 1].

### Performance metrics results

#### Theme 1: complete case-mix shift

Scenarios Complete Case-Mix/High Target and Complete Case-Mix/Low Target introduce a case-mix shift in the age variable with full separation, while maintaining other variables in the null case without shift.

**Fig. 1:**
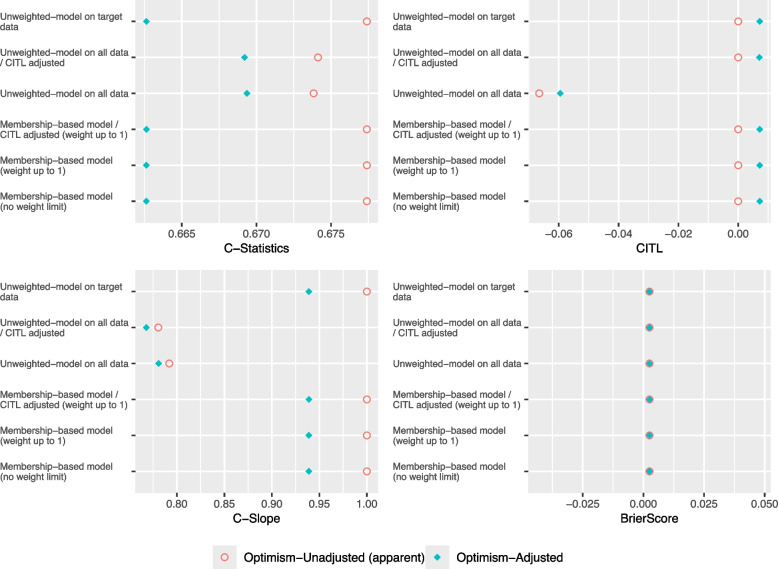
Performance Metrics for Theme 1 - Scenario 1: Complete Case-Mix/High Target

**Fig. 2:**
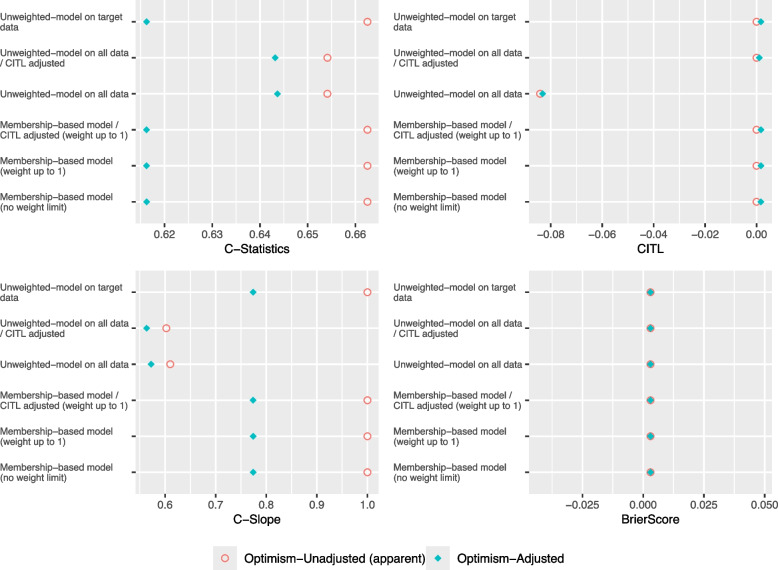
Performance Metrics for Theme 2 - Scenario 2: Complete Case-Mix/Low Target

**Fig. 3:**
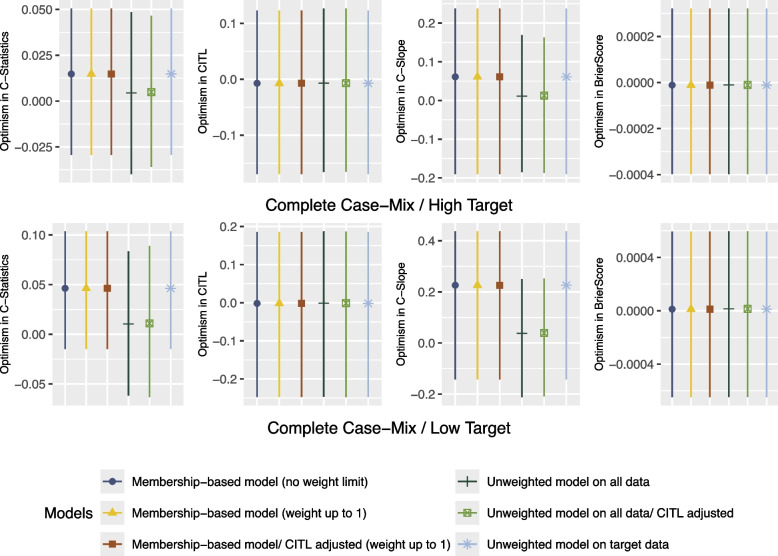
Confidence Intervals of Bootstrap Optimism for Theme 1

As shown in Figs. 1 and 2, the C-Slope and the C-Statistic for all models deteriorates in Complete Case-Mix/Low Target compared to Complete Case-Mix/High Target. The Membership-based model- CITL adjusted and the Unweighted model on target data performed exactly the same in both scenarios and achieved the closest optimism-adjusted C-Slope to 1. Furthermore, as illustrated in Fig. 3, in both scenarios, the mean optimism in C-Statistic and optimism in C-Slope of the Unweighted model on all data, with and without adjusting the CITL was closer to zero compared to Membership-based model and the Unweighted model on target data. In addition, the Unweighted model on all data, with and without adjusting the CITL showed less variability in optimism in C-Slope compared to other models.

#### Theme 2: partial case-mix shift

Scenarios Partial Case-Mix/Low Target and Partial Case-Mix/High Target introduce a case-mix shift in the age variable with partial separation, while maintaining other variables in the null case without shift.

**Fig. 4:**
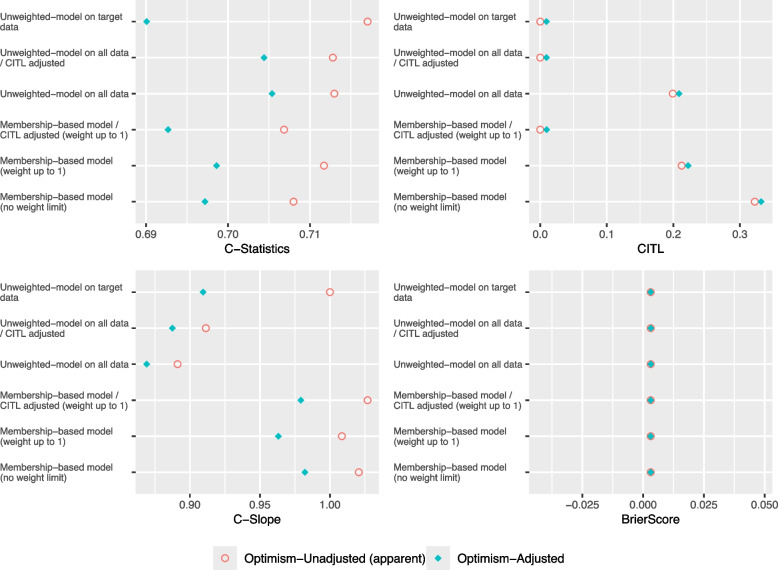
Performance Metrics for Theme 2 - Scenario 3: Partial Case-Mix/Low Target

**Fig. 5:**
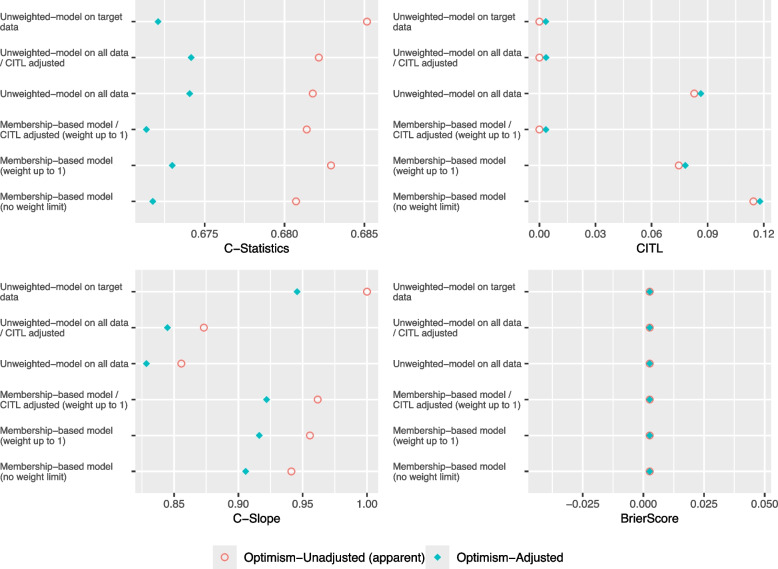
Performance Metrics for Theme 2 - Scenario 4: Partial Case-Mix/High Target

As shown in Figs. 4 and 5, in both scenarios, the Unweighted model on all data with and without CITL adjustment achieved the highest optimism-adjusted C-Statistic. Furthermore, the Membership-based models achieved the closest optimism-adjusted C-Slope to 1 in Partial Case-Mix/Low Target compared to the Unweighted models. In terms of optimism-adjusted C-Slope, the Membership-based model (no weight limit)- the baseline propensity score weighting- performed similarly to the Membership-based model with limiting the weights up to 1 and adjusting the CITL. However, the former model CITL was miscalibrated, the effective sample size was artificially inflated- see Table 4, and the standard errors of the model’s coefficients were overoptimistic. Please refer to the supplementary materials for standard errors of all models’ coefficients for all scenarios [see Additional file 1].

**Fig. 6:**
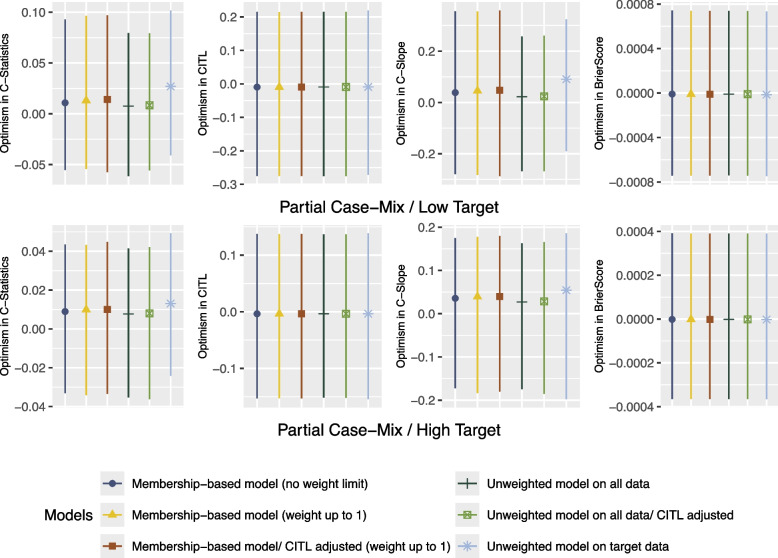
Confidence Intervals of Bootstrap Optimism for Theme 2

In scenario Partial Case-Mix/High Target where the sample size of the target set was sufficient, the Unweighted model on target data achieved the closest C-Slope to 1. Moreover, the Membership-based model with CITL adjustment and weight limited up to 1 achieved higher C-Slope compared to the model that is based on the baseline propensity score weighting without CITL adjustment. Furthermore, as illustrated in Fig. 6, in both scenarios, the mean optimism in C-Statistic and optimism in C-Slope of the Unweighted model on target data were higher compared to the other models.

#### Theme 3: insufficient target set (no case-mix shift)

Scenarios 5 Insufficient Target/Low Target and 6 Insufficient Target/Medium Target test the case when target dataset sample size is not sufficient to develop the models on, with fixed sample size for the source dataset under no case-mix shift. Also, scenario 7 Insufficient Target/High Target is included to provide a comparison between scenarios with insufficient target dataset sample sizes and a scenario where the target dataset sample size is sufficient.

**Fig. 7:**
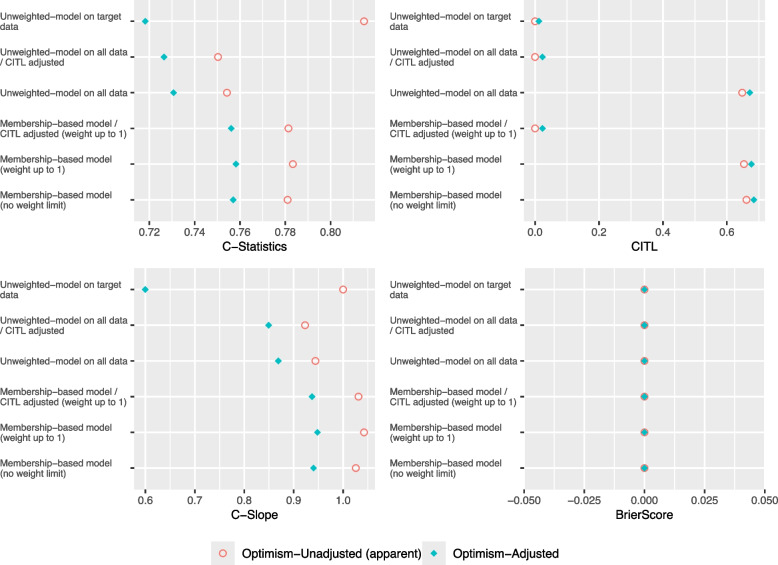
Performance Metrics for Theme 3 - Scenario 5: Insufficient Target/Low Target

**Fig. 8:**
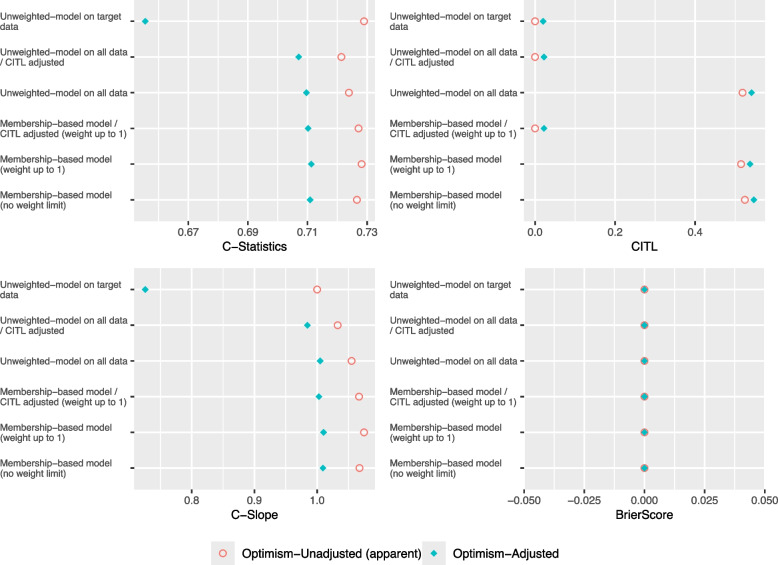
Performance Metrics for Theme 3 - Scenario 6: Insufficient Target/Low Target

As illustrated in Figs. 7 and 8 of scenarios 5 and 6, the optimism-adjusted C-Slope of the Unweighted logistic model on all data and is less than 0.9 which indicates that the model is overfitted due to developing the model with insufficient sample size, compared to scenario 7 (see Additional file 1) where the optimism-adjusted C-Slope is above 0.9 as the model is developed with sufficient sample size. The proposed Membership-based model achieved higher optimism-adjusted C-Statistic and closer optimism-adjusted C-Slope to 1 compared to the other models in all scenarios except for scenario 7 Insufficient Target/High Target where the Unweighted logistic model on target data achieved the highest C-Statistic.

**Fig. 9:**
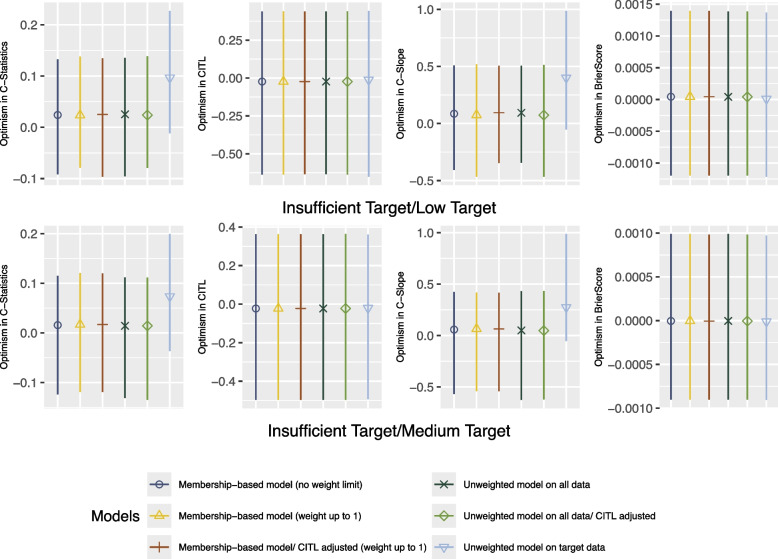
Confidence Intervals of Bootstrap Optimism for Theme 3

Furthermore, as illustrated in Fig. 9, in both scenarios the mean optimism in C-Statistic and C-Slope of the Membership-based model was closer to zero compared to the Unweighted model on target data. Moreover,it showed less variability in optimism in C-Statistic and C-Slope compared to the Unweighted model on target data in all scenarios, except for scenario 7 (see supplementary material) where the target sample size was sufficient to build the model on.

Please refer to the supplementary materials for complete results of all themes and scenarios including theme 4, bootstrap performance metrics’ confidence intervals for each scenario including C-Statistic, C-Slope, CITL, Brier Score and AUC-PR; bootstrap optimism confidence intervals in each performance metric for all models; and adjusted and unadjusted-optimism performance metrics for all models [see Additional file 1].

## Discussion

We proposed a Membership-based method that accounts for case-mix shift when developing a CPM. The method re-weights the development dataset samples to correct for distributional changes using samples relatedness to the target population; considering that the latest distribution shift in the development dataset reflects the distribution of the target population. Booth et al. [21] had similar consideration in the temporal recalibration approach, which involves developing a model in the entire dataset and then recalibrates the baseline survival using a period analysis sample. Thus, providing more up-to-date survival predictions that more closely match the observed survival in subsequent data than the standard full cohort models.

We proposed to define relatedness based on the conditional probability of the sample being a member of the target population using membership propensity score. Furthermore, we extend the baseline propensity score by 1) Exploiting the shift within the development dataset itself to account for the case-mix shift between the development and deployment phases without requiring additional or external data samples. 2) Multiplying the propensity score by the sample size ratio. 3) Controlling the effective sample size of the source set by limiting the weights up to one or applying a scaling factor. 4) Recalibrating the prediction model after weighting by adjusting the calibration-in-the-large. We compare the proposed method with the baseline Propensity Score/Importance Weighting [44–46] that entails estimating the density ratio between the target data distribution and the source data distribution, followed by weighting the source data using this ratio. However, the baseline method artificially inflates the effective sample size of the model and generates overoptimistic standard errors of the model’s coefficients. Moreover, we compare the proposed method with two existing CPM development methods, the first develop the model on all data, and the second develop the model on the target data only that has the most recent change [18]. However, these methods are inefficient as the former does not account for the distributional changes, while the latter results in a reduction of sample size making the model prone to overfitting [21, 22].

Similar work in transportability literature was proposed to transport a prediction model from one population (source) to a new target population under the case-mix shift using propensity score as a balancing score [47–50]. Steingrimsson et al. [47] proposed to weight the maximum likelihood estimator using the inverse-odds weights obtained from estimating the probability of membership in the source population, using training data from the source population and unlabeled data from target population. Similarly, Autenrieth et al. [48] proposed to partition (stratify) the data into subgroups to condition on propensity scores to avoid the massive variance associated with importance weighting. However, the applicability of these two methods [47, 48] may be limited by the lack of access to additional new data samples from the target population and the assumption that *P*(*Y*|*X*) remains unchanged. In transportability of randomized controlled trials (RCTs), Chu et al. and Chen et al. [51, 52] proposed a weighting approach based on the summary-level information from the target sample to account for covariate shift by balancing the covariates using propensity score. However, there are significant differences between transportability analyses for causal effects compared prediction models.

Others studied the external validity of risk prediction model in target population [53–55]. Pfeiffer et al. [55] and De Jong et al. [54] proposed to use propensity-weighted measures of discrimination that adjust for differences in the covarites distributions between the training and validation datasets. Related ideas have also appeared in literature on accounting for case-mix shift involving updating, refitting, or recalibrating [5]. Davis et al. proposed a testing procedure to recommend updating methods to maintain CPMs performance over time [27].

We report the following findings:

- *Theme 1- Complete case-mix shift:* In both scenarios where the shift brings forth completely new values, there is an increasing need to down-weight the pre-shift data samples (data samples before the shift happen). This functionality is effectively offered by the proposed Membership-based method as the Unweighted model on all data achieved the lowest optimism-adjusted C-Slope compared to all other models. Furthermore, in both scenarios, the model developed on target data and the Membership-based model had identical performance, as the latter down-weighted all the source data samples near to zero.
- *Theme 2- Partial case-mix shift:* In both scenarios where the shift brings forth partially new values (the data has not completely shifted), there is a need to use the proposed weighting method. Particularly, when the target data is not of sufficient sample size to develop the model as the Membership-based model achieved the highest optimism-adjusted C-Slope in scenario 3. However, when the target data is of sufficient sample size to develop the model, the Unweighted model on the target data only achieved the highest optimism-adjusted C-Slope.
- *Theme 3 and 4- No case-mix shift:* The proposed Membership-based method showed robustness to work under no case-mix shift (the null case). In scenarios 5, 6, 7 and 8, the proposed Membership-based method achieved better performance, in terms of optimism-adjusted C-Slope, compared Unweighted model on target data only and Unweighted model on all data/CITL adjusted. Moreover, in scenarios 5 and 6 where the target data was not of sufficient sample size to develop the model, the Membership-based method achieved better performance, in terms of optimism-adjusted C-Statistic compared to the other methods. [See Additional file 1 for the full results of themes 3 and 4].

### Study strength and limitations

Accounting for case-mix shift in the development dataset may improve the model fit for a given target dataset by incorporating data from the source dataset. This study has several strengths. First, The proposed method re-weights individual samples based on similarity, which helps in identifying and giving importance to individuals who are more relevant to the target data, thus, effectively addresses the problem of sample size reduction by utilising all the available data. Particularly, when target dataset is not of sufficient sample size to develop the model. Moreover, the proposed method enables faster adaptation to distributional changes in the target data, over time or over space. This is particularly valuable in dynamic environments with case-mix shift presents in the development dataset which can impact model performance. Finally, the proposed method demonstrates robustness to both case-mix shift and general applicability under null cases, making it suitable for a wide range of scenarios. Since certainty about the presence or absence of a shift is never guaranteed in practice, the method can be applied without incurring any penalties. Nevertheless, several important limitations must be addressed.

First, this study only considers the case-mix shift and the null case of no case-mix shift in the development dataset. However, there might be an effect for other types of data distribution shift, such as predictor-outcome shift, which the proposed method does not account for. This could be tackled by modifying the proposed approach to mitigate the predictor-outcome shift. In addition, the applied CITL adjusting method to crudely account for the event rate shift might not be the best method to apply as adding a dummy variable in the model changes the number of predictors and level of overfitting of the model, hence affect the C-Slope. Improvements to the crude adjustment could be considered by including the outcome *Y* in the weighting schemes of the proposed method, or by applying a two-steps approach.

Another limitation of this study is the use of a single dataset in the case study. While the dataset provided valuable insights into the specific context and scenarios under investigation to explore the properties of the proposed method, it raises questions about the generalisability of the findings to broader populations or different settings and scenarios. Future work should aim to replicate and validate these findings across different simulated and real-world datasets to create a more robust foundation for generalisability (phase three of methodological research study [29]). Finally, the estimate of propensity score in the Membership-based approach with logistic model might not be efficient; higher order terms and interactions could be considered.

## Conclusion

The proposed method shows promising results in accounting for the case-mix shift in the development dataset by using all available data and prior knowledge. We recommend using the Membership-based weighted model when case-mix shift is partially separating the values between source and target dataset, especially in cases where the target data is not of sufficient sample size to develop the model. Furthermore, we recommend using the Membership-based weighted model or developing the model using the target data only when case-mix shift is completely separating the values between source and target datasets. Nonetheless, it is important to note that in our experiments, we had prior knowledge of the presence or absence of a case-mix shift. In practical scenarios, such certainty never exists. Therefore, we recommend consistently applying the proposed method, as it has demonstrated robustness even in null cases. Thus, we can anticipate potential benefits, no matter how modest, without incurring any penalties. Further investigation and testing are needed, as well as accounting for predictor-outcome association shift.

## Supplementary Information

Additional file 1: Section 1. THEMES AND SCENARIOS: Further details on scenarios’ datasets splitting. Section 2. ADJUSTED OPTIMISM ESTIMATION: Illustration of estimating the optimism-adjusted performance metric. Section 3. EFFECTIVE SAMPLE SIZE: Results of effective sample size for the rest of scenarios. Section 4. PERFORMANCE METRICS RESULTS: Theme 1 and Theme 2 performance metrics’ results. Section 5. ADJUSTED AND UNADJUSTED-OPTIMISM PERFORMANCE METRICS RESULTS: Results of adjusted and unadjusted-optimism performance metrics for all models. Section 6. CONFIDENCE INTERVALS OF BOOTSTRAP OPTIMISM: Results of optimism confidence intervals for all models. Section 7. BOOTSTRAP PERFORMANCE METRICS: Results of bootstrap performance metrics’ confidence intervals for each scenario including C-Statistic, C-Slope, CITL, Brier Score, and AUC-PR. Section 8. MODELS’ COEFFICIENTS STANDARD ERRORS: Standard errors of all models’ coefficients for all scenarios.

## Data Availability

The datasets analysed during the current study are not publicly available due to the required data sharing agreements and considerations. Access to data Available for research by application to the SWEDEHEART steering group. All statistical code for the analysis is available through open-source repositories (GitHub) for full transparency and reproducibility.

## Abbreviations

CPM: Clinical Prediction Model
C-slope: Calibration slope
SWEDEHEART: The Swedish Web-System for Enhancement and Development of Evidence-Based Care in Heart Disease Evaluated According to Recommended Therapies
ACS: Acute coronary syndrome
ICD: Implantable Cardioverter-Defibrillator
MI: Myocardial infarction
OHCA: Out-of-hospital cardiac arrest
AF: Atrial fibrillation/flutter
BMI: Body mass index
LVEF: Left ventricular ejection fraction
eGFR: Estimated glomerular filtration rate
CKD-EPI: Chronic Kidney Disease Epidemiology Collaboration
CITL: Calibration in the large
ROC: Receiver operating characteristic
AUC-PR: Area under the precision and recall curve
RCTs: Randomized controlled trials
