# Supplementary material for: Correcting for case-mix shift when developing clinical prediction models
Source: BMC Med Res Methodol. 2025 Aug 1;25:186. doi: 10.1186/s12874-025-02621-2 (PMC12317593; doi:10.1186/s12874-025-02621-2)
Supplement: Supplementary file 1 — Additional file 1: Section 1. THEMES AND SCENARIOS: Further details on scenarios’ datasets splitting. Section 2. ADJUSTED OPTIMISM ESTIMATION: Illustration of estimating the optimism-adjusted performance metric. Section 3. EFFECTIVE SAMPLE SIZE: Results of effective sample size for the rest of scenarios. Section 4. PERFORMANCE METRICS RESULTS: Theme 1 and Theme 2 performance metrics’ results. Section 5. ADJUSTED AND UNADJUSTED-OPTIMISM PERFORMANCE METRICS RESULTS: Results of adjusted and unadjusted-optimism performance metrics for all models. Section 6. CONFIDENCE INTERVALS OF BOOTSTRAP OPTIMISM: Results of optimism confidence intervals for all models. Section 7. BOOTSTRAP PERFORMANCE METRICS: Results of bootstrap performance metrics’ confidence intervals for each scenario including C-Statistic, C-Slope, CITL, Brier Score, and AUC-PR. Section 8. MODELS’ COEFFICIENTS STANDARD ERRORS: Standard errors of all models’ coefficients for all scenarios. [file 12874_2025_2621_MOESM1_ESM.pdf]

1 | THEMES AND SCENARIOS

Theme 1 introduces a case-mix shift in one continuous variable in the dataset, while maintaining the other variables in the null case without shift. In scenario 1 (**Complete Case-mix/High Target** and scenario 2 **Complete Case-mix/Low Target**), the development dataset is split into source and target datasets by completely separating the age variable, where the source set consists of all individuals with the ages above 75 and the target set consists of all individuals with the ages below 75, without considering the time factor. Scenario **Complete Case-mix/Low Target** has insufficient sample size for the target dataset compared to **Complete Case-mix/High Target** that achieves the minimum sample size criteria for the target dataset. Theme 2 introduces a case-mix shift in one continuous variable in the dataset, while maintaining the other variables in the null case without shift. In scenario 3 (**Partial Case-mix/Low Target** and scenario 4 **Partial Case-mix/High Target**) the age variable was partially separated to have an overlap between the source set that contains ages between [18, 80] and target set that contains ages between [57, 103]. In Theme 1 and 2, the split in the development dataset into source and target sets by age cut-off was inspired from the shift in the age mean in EuroSCORE prediction model. The shift could also represent data from different domains, such as patients from different healthcare settings (e.g., primary versus secondary care), inpatients versus outpatients, or varying age categories (e.g., adults versus children). These domains often demonstrate differences in patient case mix, reflected in variations in the distribution and range of predictor values. Table S1 shows the selection of the sets in more details and the themes and scenarios' details.

Theme 3 contains two scenarios (Scenario 5 **Insufficient Target/Low Target**, Scenario 6 **Insufficient Target/Medium Target**) to test the case when the target set sample size is not sufficient (alone) to develop the models on, with fixed sample size for the source dataset. Also, scenario 7 **Insufficient Target/High Target** is included to provide a comparison between scenarios with insufficient target dataset sample sizes and a scenario where the target dataset sample size is sufficient. The development dataset is split into source and target datasets based on calender time, where the target set is representing the most recent data. The split was chosen to effectively demonstrate the methodology, rather than based on an intrinsic property of the data. In practice, a dichotomy of time used to define target and source datasets should be informed by the clinical context, such as changes in clinical guidelines, changes in clinical coding systems, changes in underling patient population. This approach can also be useful in scenarios where separate datasets are available—for instance, a large source dataset and a smaller target dataset—making the distinction more a function of data availability than population differences. Additionally, it may be relevant in situations involving shock to the system, such as COVID-19 happening suddenly, or in cases of transfer learning, where a model initially developed for one condition, such as pneumonia, is adapted for another, such as COVID-19, as frequently occurred during the early stages of the pandemic16. The target dataset for **Insufficient Target/Low Target** is selected from a random sample from 2017, and for **Insufficient Target/Medium Target** the target dataset is selected from years 2016 and 2017. Furthermore, this theme is not introducing a case-mix shift within the dataset, thus, it tests the approaches under the null case (case of no case-mix shift).

Theme 4 contains three scenarios to test the usefulness of borrowing strength from the source data at different rates with a fixed sample size for the target dataset. This theme is not introducing a case-mix shift within the dataset, thus, it tests the approaches under the null case (case of no case-mix shift). We also considered the calculated minimum required sample size for the development dataset to define the target set in scenarios 7,8 and 9. For scenario 8 (**Borrow Strength/Low Source**) the source dataset is selected from years 2006 to 2008 and 33,123 random samples are selected from years 2015 to 2017 for the target dataset. For scenario 7 (**Borrow Strength/Medium Source, Insufficient Target/High Target**) the source dataset is selected from years 2006 to 2010 and 33,123 random samples are selected from years 2015-2017 for the target dataset. Finally, for scenario 9 (**Borrow Strength/High Source**) the source dataset is selected from years 2006 to 2014 and 33,123 random samples are selected from years 2015 to 2017 for the target dataset.

Table S1 Themes and Scenarios' Details

Theme	Scenario	Source set selection & sample size	Target set selection & sample size	Total sample size	Is Target set of sufficient sample size?	Source set proportion	Target set proportion
Theme 1: Complete case-mix shift	1) Complete Case-Mix / Low Target	Sample of size 92734 of all individuals with ages between [18, 80]	Random sample of size 20000 of individuals with ages between [57, 103]	112734	Not sufficient	82%	18%(low)
Theme 1: Complete case-mix shift	2) Complete Case-Mix / High Target	Sample of size 92734 of all individuals with ages between [18, 80]	Random sample of size 73660 of individuals with ages between [57, 103]	166394	Sufficient	56%	44%(high)
Theme 2: Partial case-mix shift	3) Partial Case-Mix / High Target	Sample of size 98716 of all individuals with ages >75	Random sample of size 67678 of individuals with ages <= 75	166394	Sufficient	59%	41%(high)
Theme 2: Partial case-mix shift	4) Partial Case-Mix / Low Target	Sample of size 98716 of all individuals with ages >75	Random sample of size 20000 individuals with ages <= 75	118716	Not sufficient	83%	17%(low)
Theme 3: Insufficient Target set (no case-mix shift)	5) Insufficient Target/Low Target	Sample of size 70269 of all individuals from years 2006 to 2010	Random sample of size 5000 from year 2017	75269	Not sufficient	93%	7% (low)
Theme 3: Insufficient Target set (no case-mix shift)	6) Insufficient Target/Medium Target	Sample of size 70269 of all individuals from years 2006 to 2011	Random sample of size 11065 of individuals from years 2016 and 2017	81334	Not sufficient	86%	14% (medium)
Theme 3: Insufficient Target set (no case-mix shift)	7) Insufficient Target/High Target	Sample of size 70269 of all individuals from years 2006 to 2012	Random sample of size 33123 of individuals from years 2015 to 2017	103392	Sufficient	68%	32% (high)
Theme 4: Borrowing strength from Source set (no case-mix shift)	8)Borrow Strength/Low Source	Sample of size 42215 of all individuals from years 2006 to 2008	Random sample of size 33123 of individuals from years 2015 to 2017	75338	Sufficient	56% (low)	44%
Theme 4: Borrowing strength from Source set (no case-mix shift)	7)Borrow Strength/Medium Source	Sample of size 70269 of all individuals from years 2006 to 2010	Random sample of size 33123 of individuals from years 2015 to 2017	103392	Sufficient	68% (medium)	32%
Theme 4: Borrowing strength from Source set (no case-mix shift)	9)Borrow Strength/High Source	Sample of size 127605 of all individuals from years 2006 to 2014	Random sample of size 33123 of individuals from years 2015 to 2017	160728	Sufficient	79% (high)	21%

2 | ADJUSTED OPTIMISM ESTIMATION

Figure S1 illustrates the approach of estimating the adjusted-optimism performance metric.

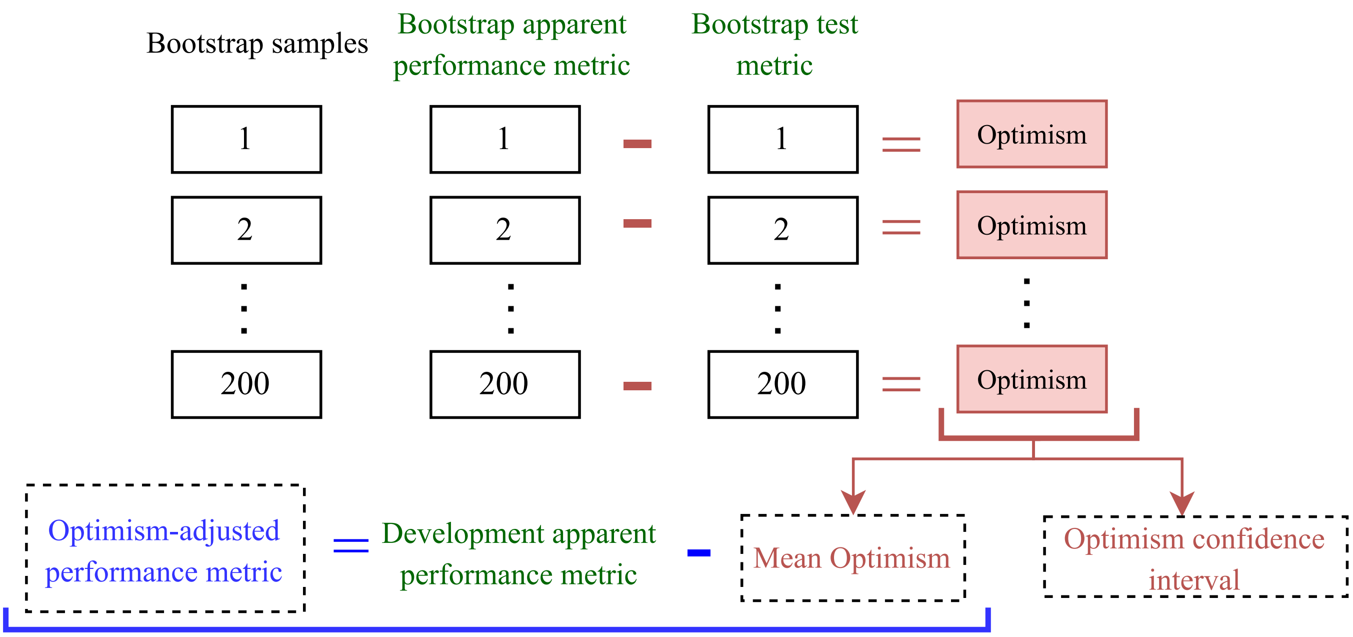

Figure S1 Adjusted optimism estimation

3 | EFFECTIVE SAMPLE SIZE

Table S2 shows the source set weights and the effective sample size for scenarios 7,8 and, 9.

Table S2 Weights and Total Effective Sample Size for Models

Scenario	Scenario 7	Scenario 8	Scenario 9
Membership-based source set weights (no weight limit)	70262	42209	127603
Membership based model effective sample size (no weight limit)	103385	75332	160726
Membership based source set weights (weight up to 1)	64954	38459	120878
Membership based model effective sample size (weight up to 1)	98077	71582	154001
Unweighted model on all data effective sample size	103392	75338	160728
Unweighted model on target data effective sample size	33123	33123	33123

4 | PERFORMANCE METRICS RESULTS

4.1 | Theme 4: Borrowing strength from the source data (no case-mix shift)

Scenarios **Borrow Strength/Low Source**, **Borrow Strength/Medium Source**, and **Borrow Strength/High Source** test the usefulness of borrowing strength from the source data at different rates with a fixed sample size for the target dataset under no case-mix shift.

As shown in Figures S9, S8, and S10, the Unweighted model on target data achieved the highest optimism-adjusted C-Statistic in all scenarios. However, in scenarios 7 and 8 - Figures S8 and S9, the proposed Membership-based model achieved the closest adjusted-optimism C-Slope to 1 compared to the unweighted models. Furthermore, as illustrated in Figure S14, in all scenarios the mean optimism in C-Statistic and C-Slope of the proposed Membership-based model were closer to zero compared to the Unweighted model on target data. However, the Unweighted model on target data showed less variability in optimism in C-Statistic compared to the other models that borrow samples from the source set. In addition, the Membership-based model showed more variability in optimism in C-Slope compared to the Unweighted model on target data and less variability compared to the Unweighted model on all data in all scenarios.

5 | ADJUSTED AND UNADJUSTED-OPTIMISM PERFORMANCE METRICS RESULTS

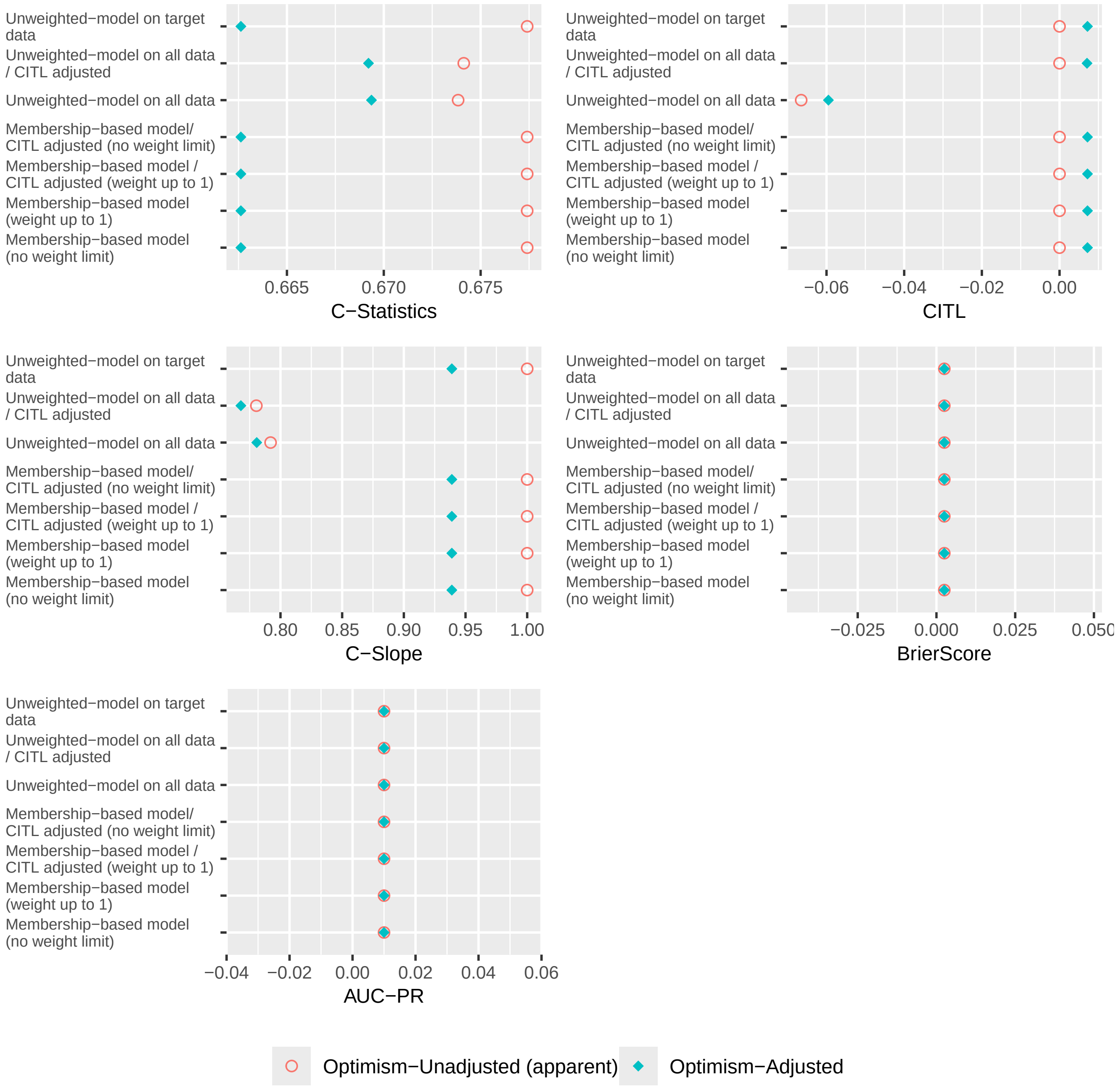

Figure S2 Performance Metrics for Theme 1- Scenario 1: Complete Case-Mix/High Target

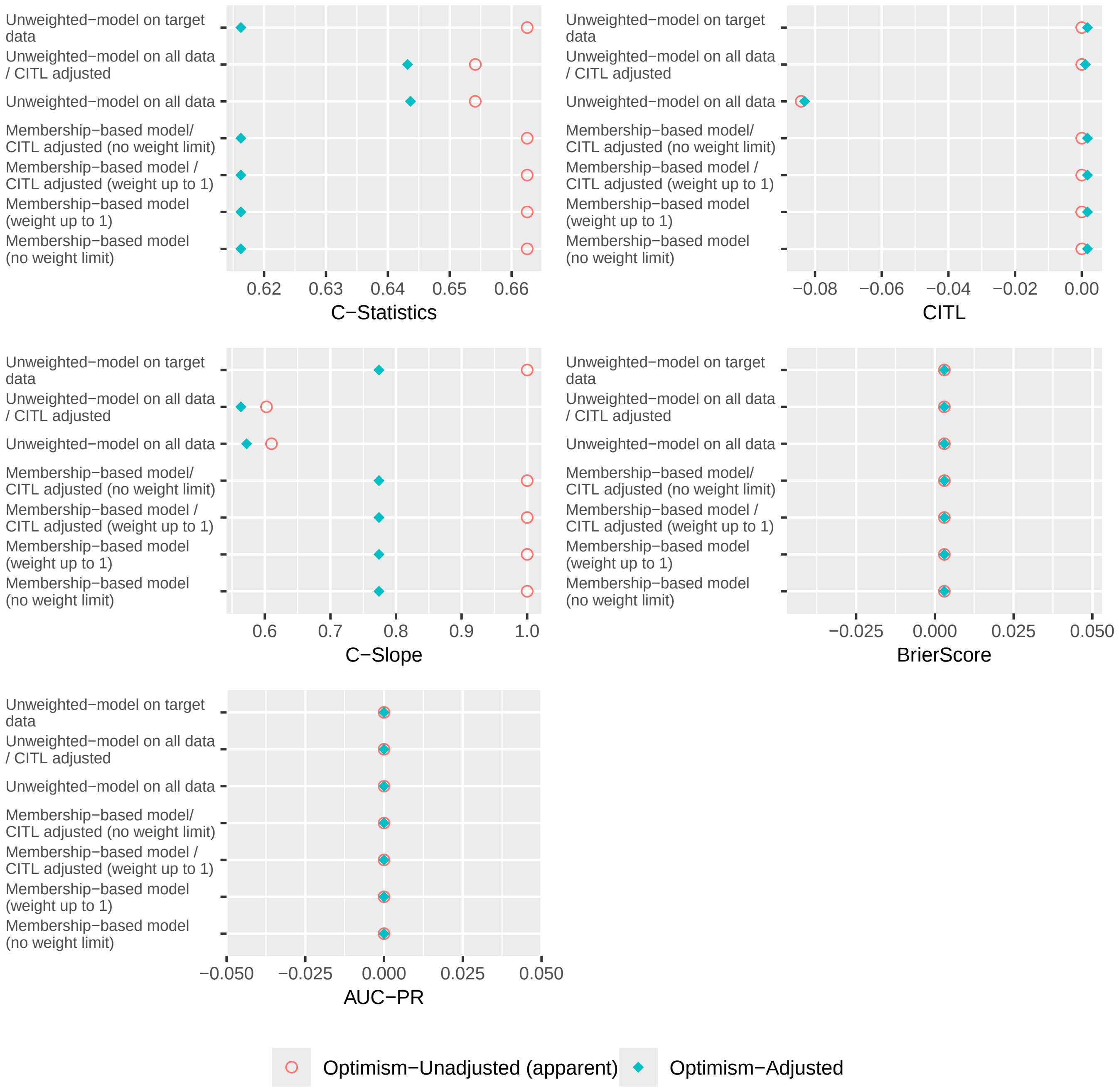

Figure S3 Performance Metrics for Theme 1- Scenario 2: Complete Case-Mix/Low Target

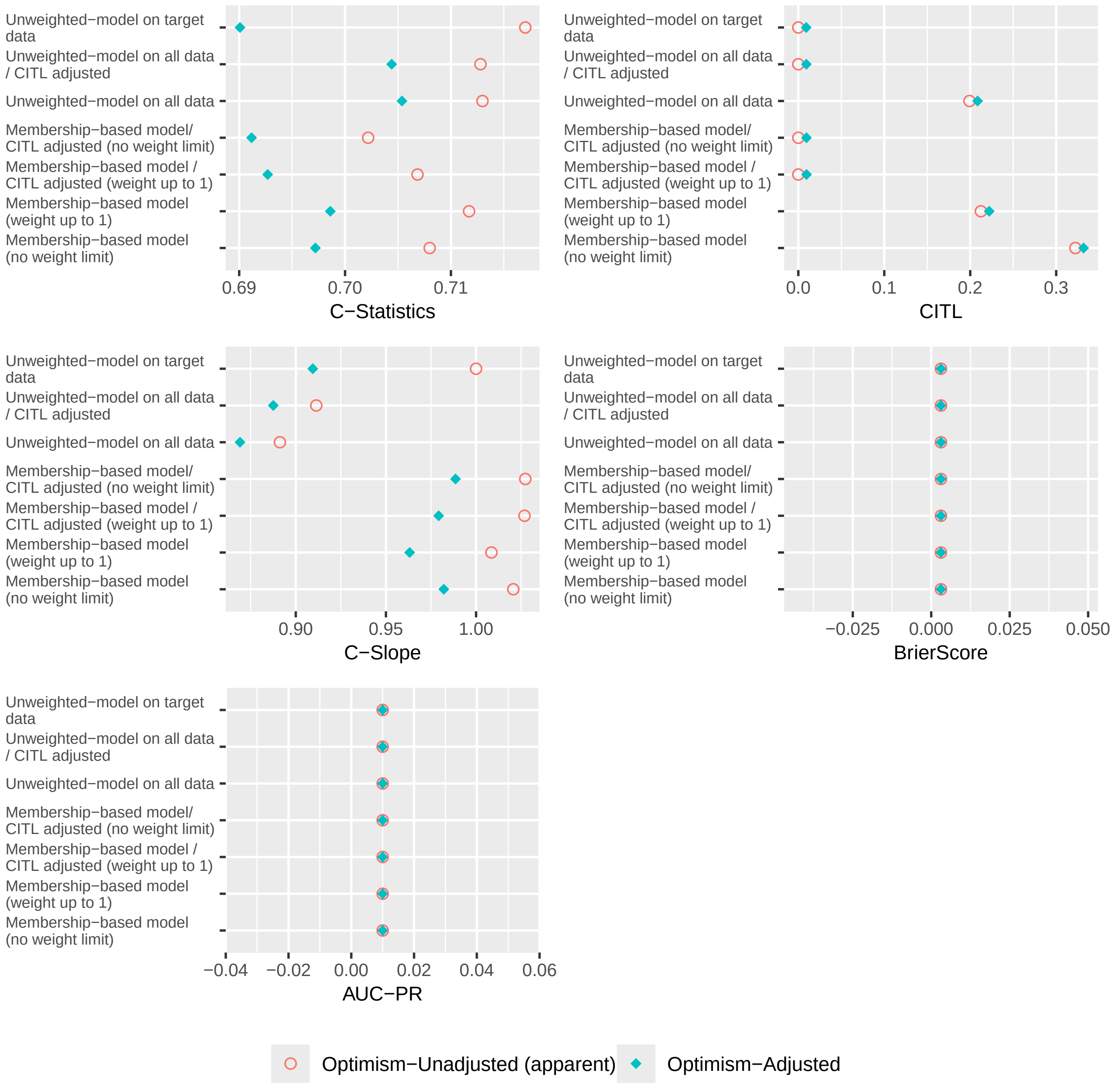

Figure S4 Performance Metrics for Theme 2- Scenario 3: Partial Case-Mix/Low Target

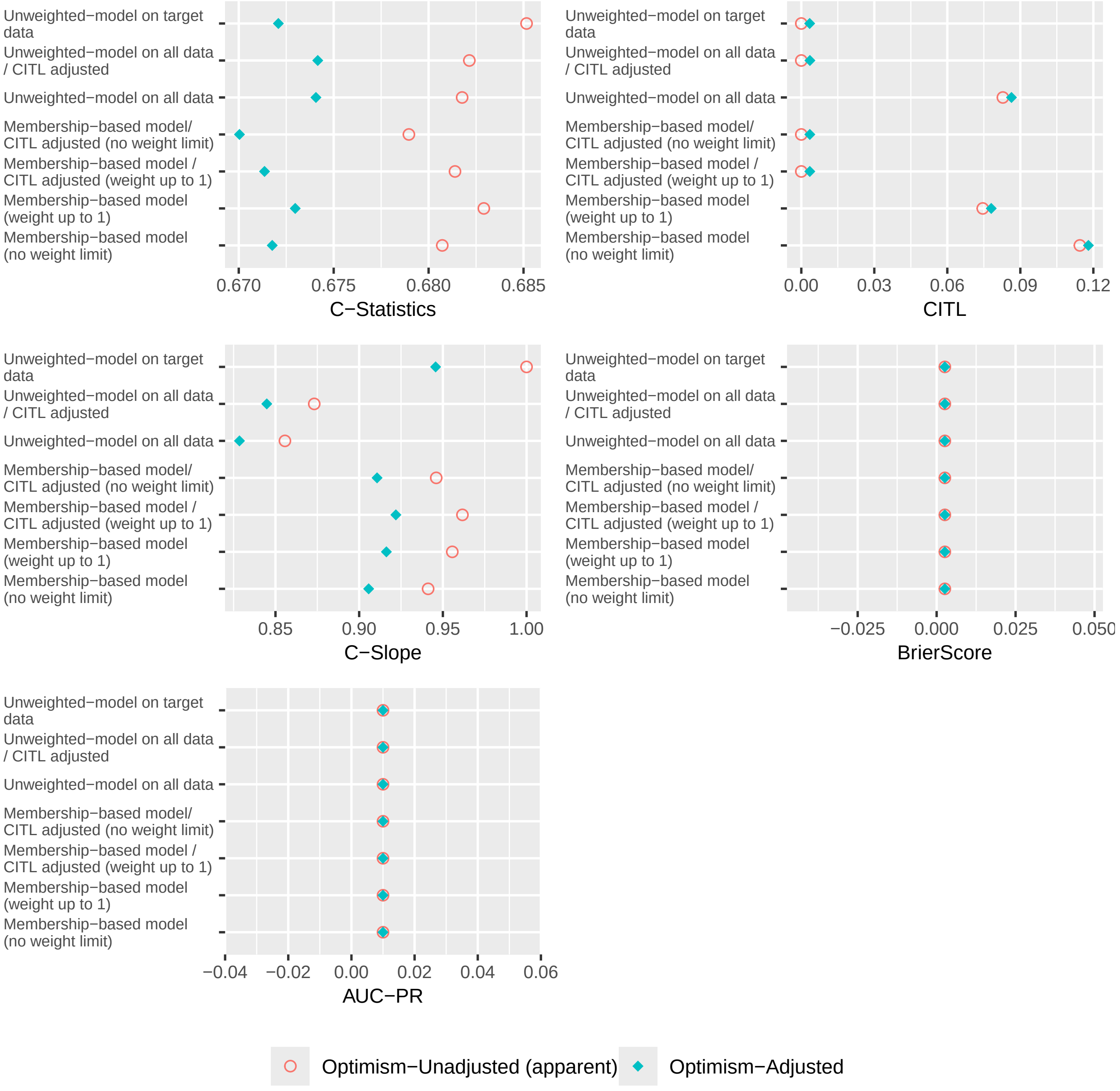

Figure S5 Performance Metrics for Theme 2- Scenario 4: Partial Case-Mix/High Target

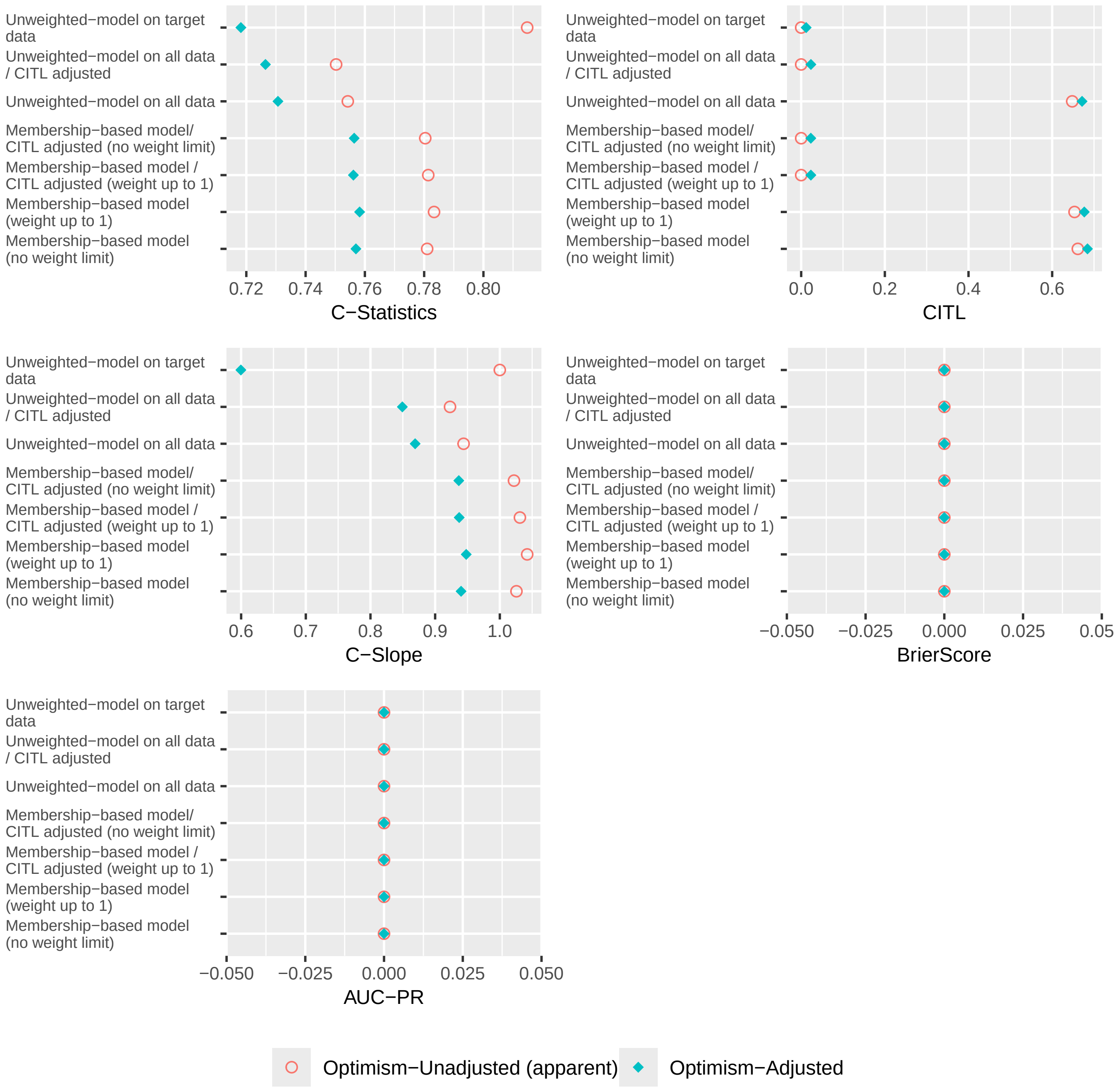

Figure S6 Performance Metrics for Theme 3- Scenario 5: Insufficient Target/Low Target

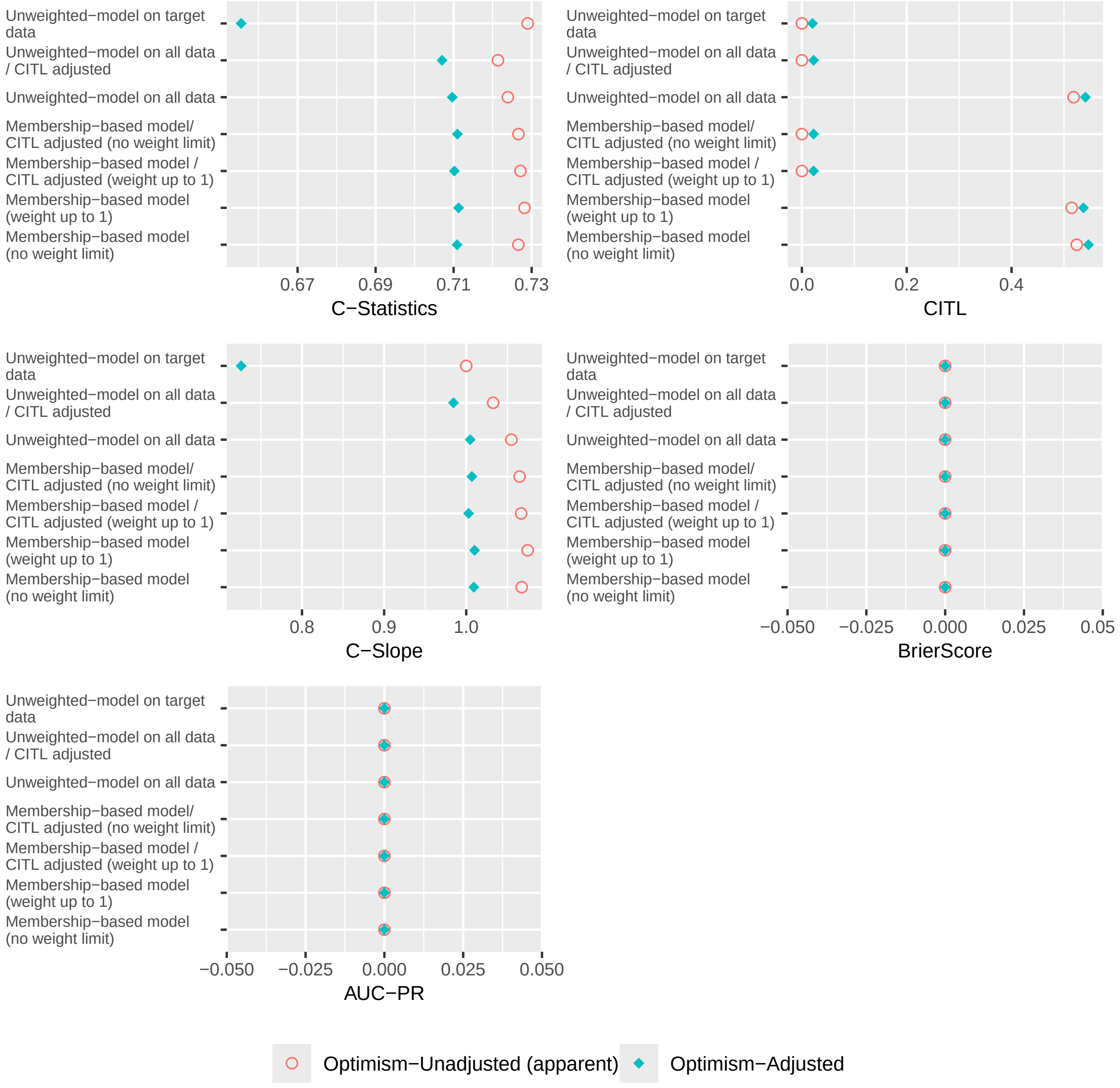

Figure S7 Performance Metrics for Theme 3- Scenario 6: Insufficient Target/Medium Target

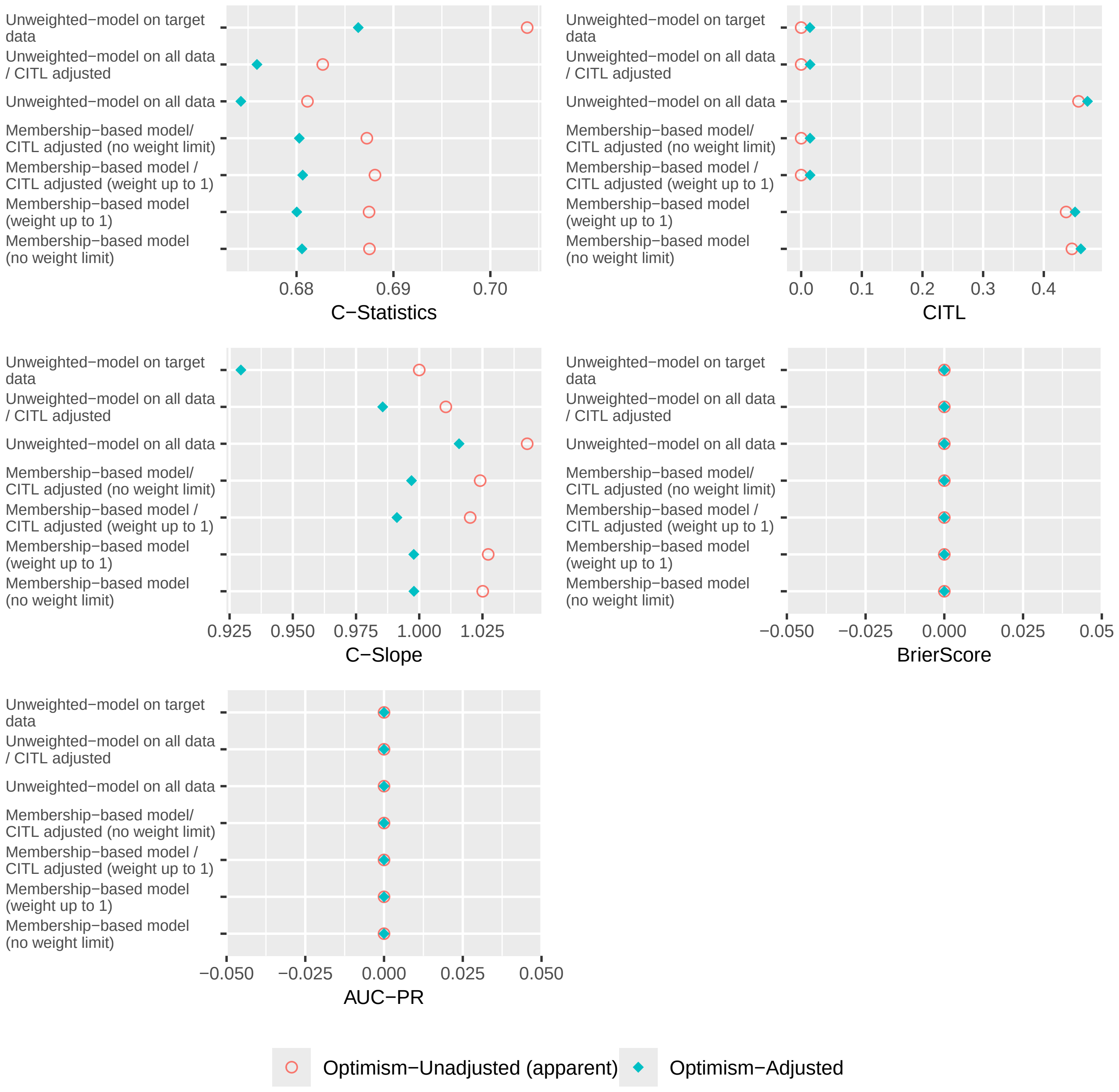

Figure S8 Performance Metrics for Scenario 7: Insufficient Target/High Target, Borrow Strength/Medium Source

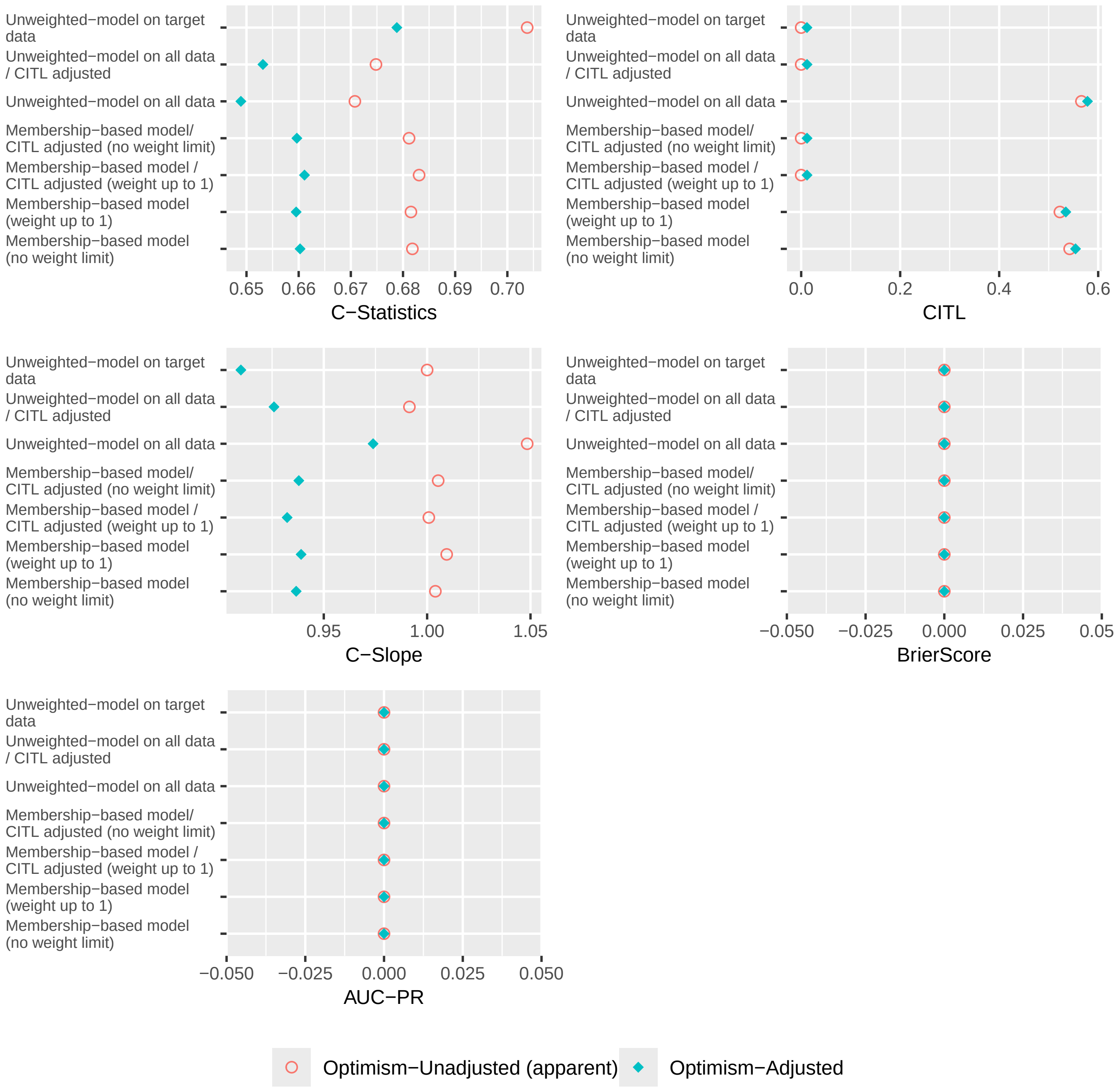

Figure S9 Performance Metrics for Theme 4- Scenario 8: Borrow Strength/Low Source

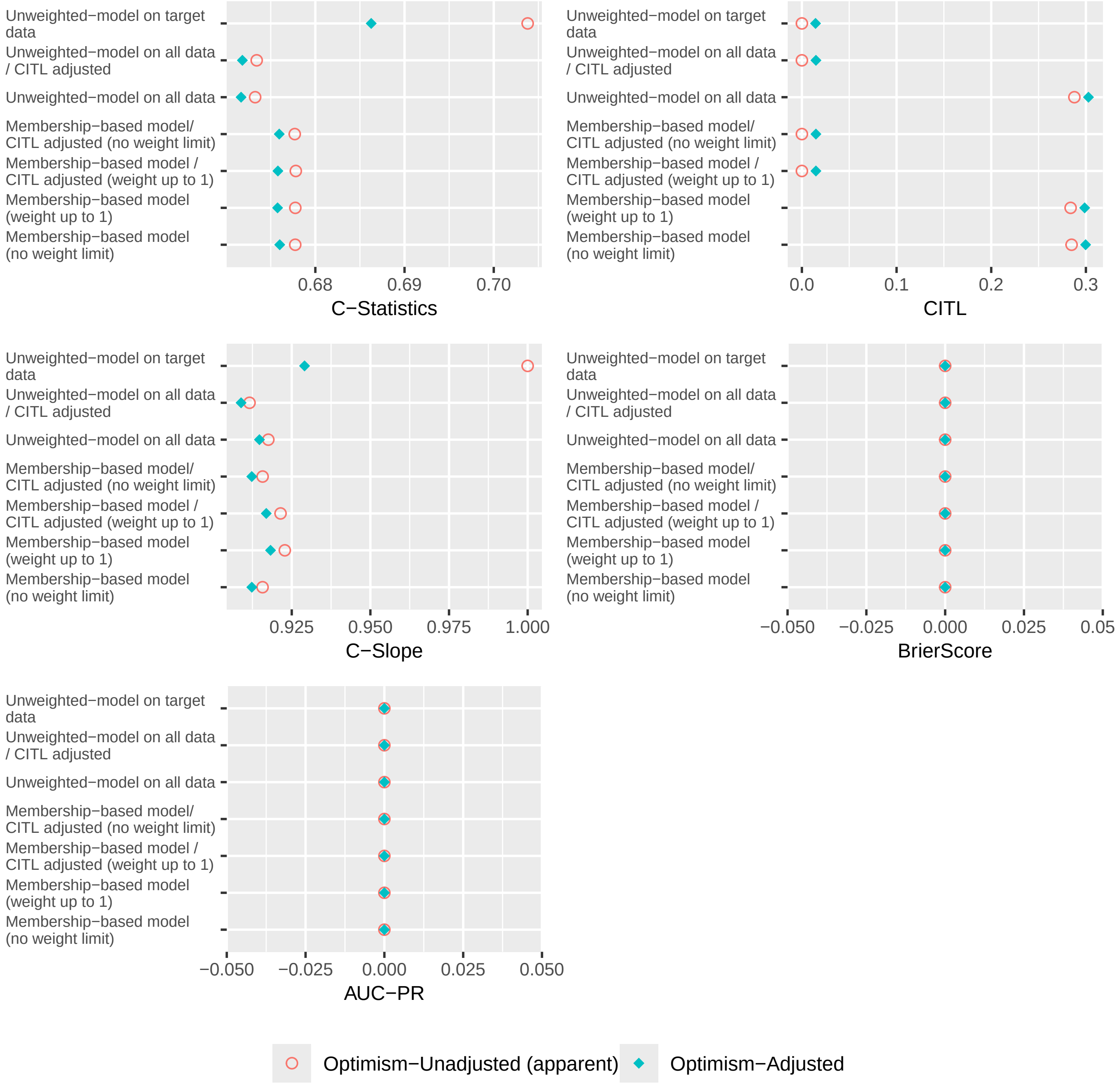

Figure S10 Performance Metrics for Theme 4- Scenario 9: Borrow Strength/High Source

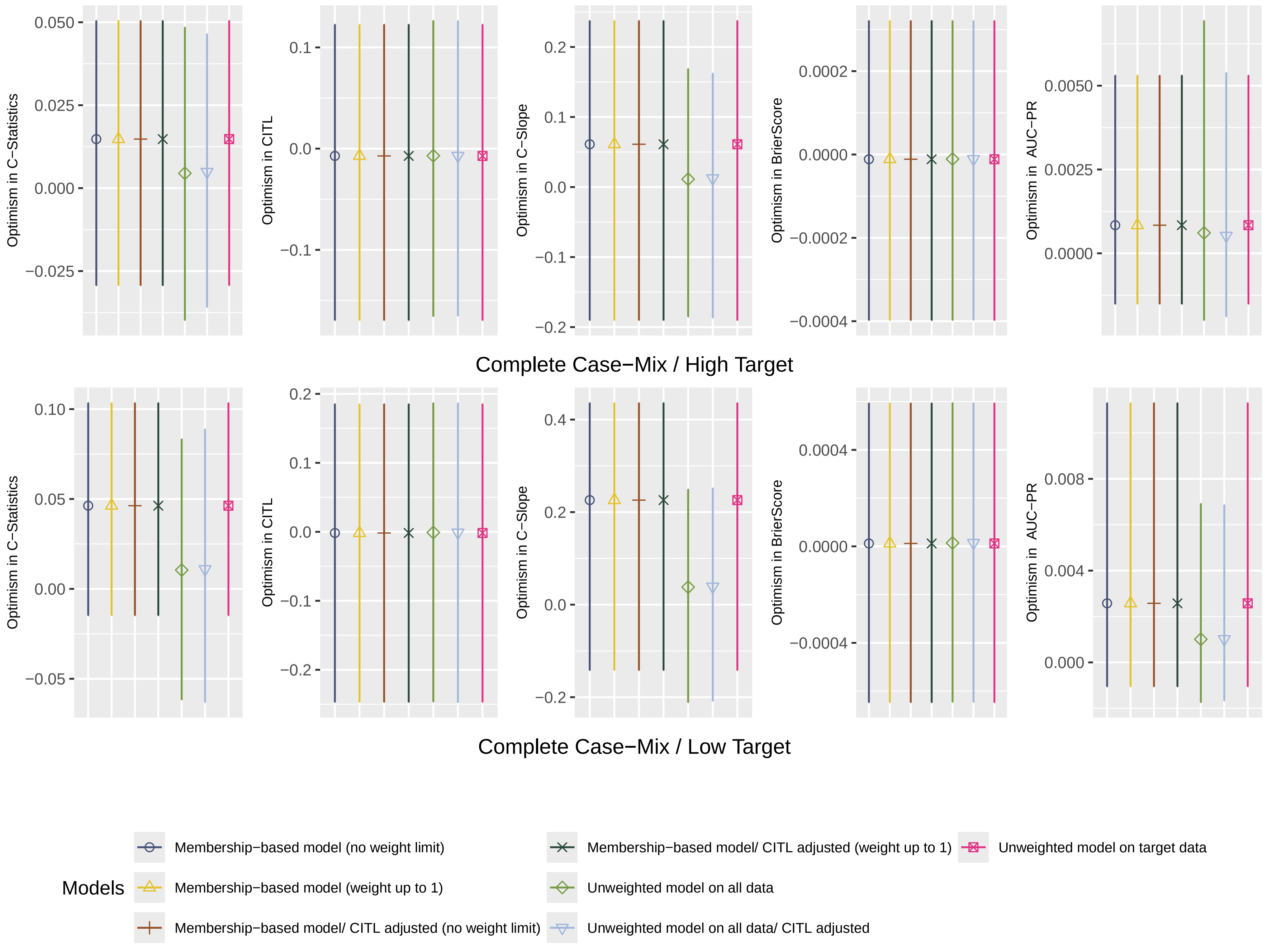

Figure S11 Confidence Intervals of Bootstrap Optimism for Theme 1

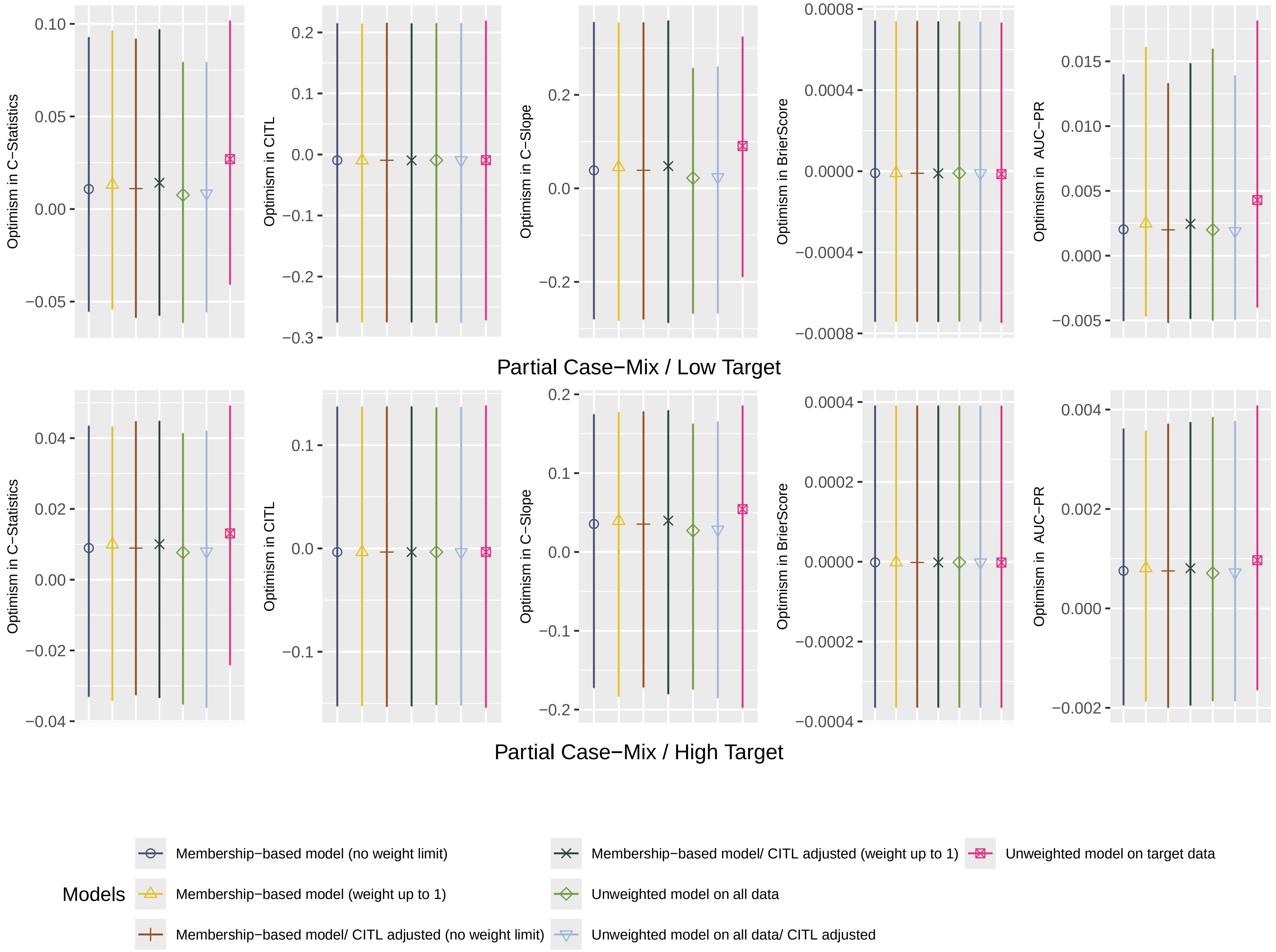

Figure S12 Confidence Intervals of Bootstrap Optimism for Theme 2

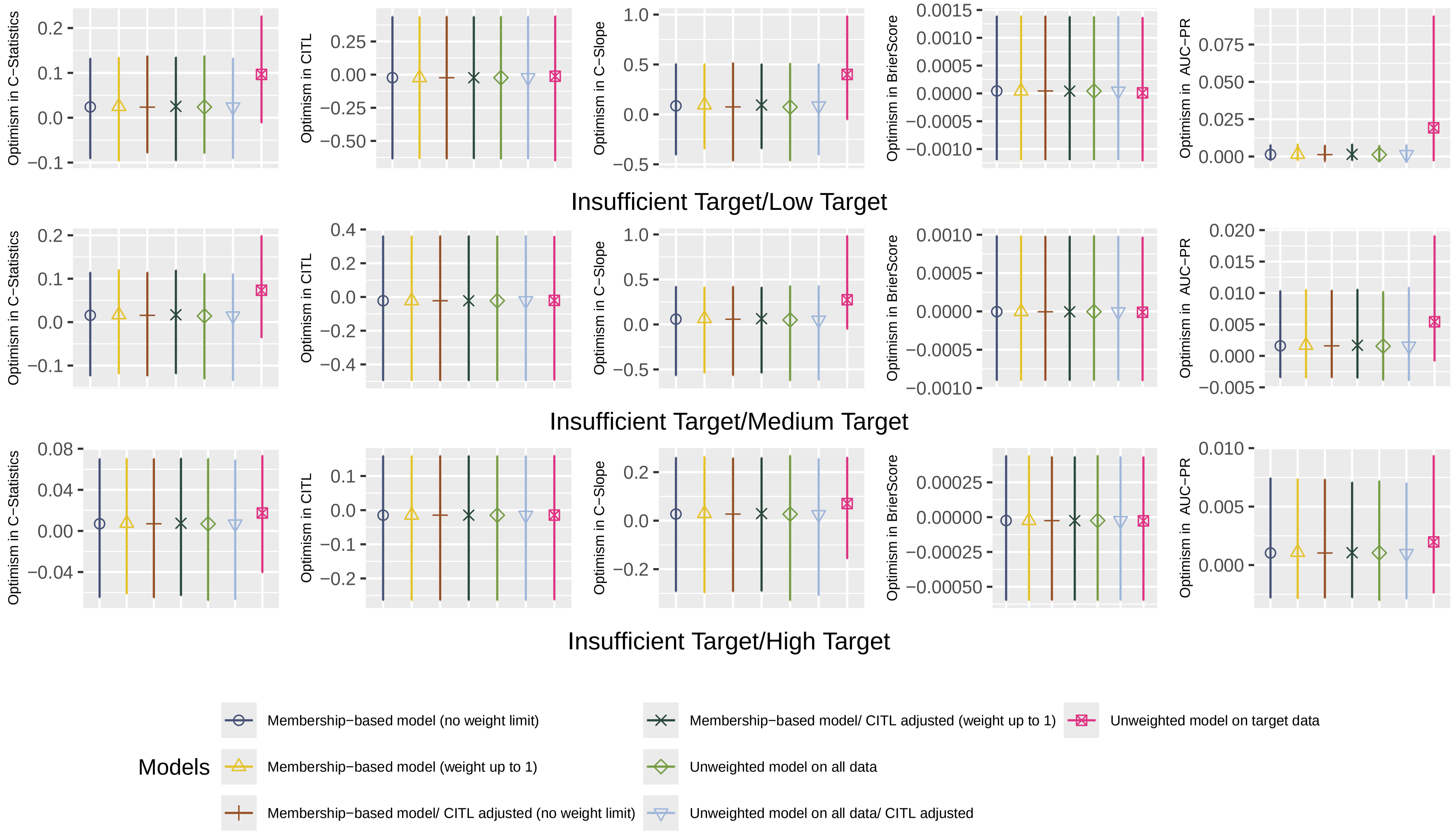

Figure S13 Confidence Intervals of Bootstrap Optimism for Theme 3

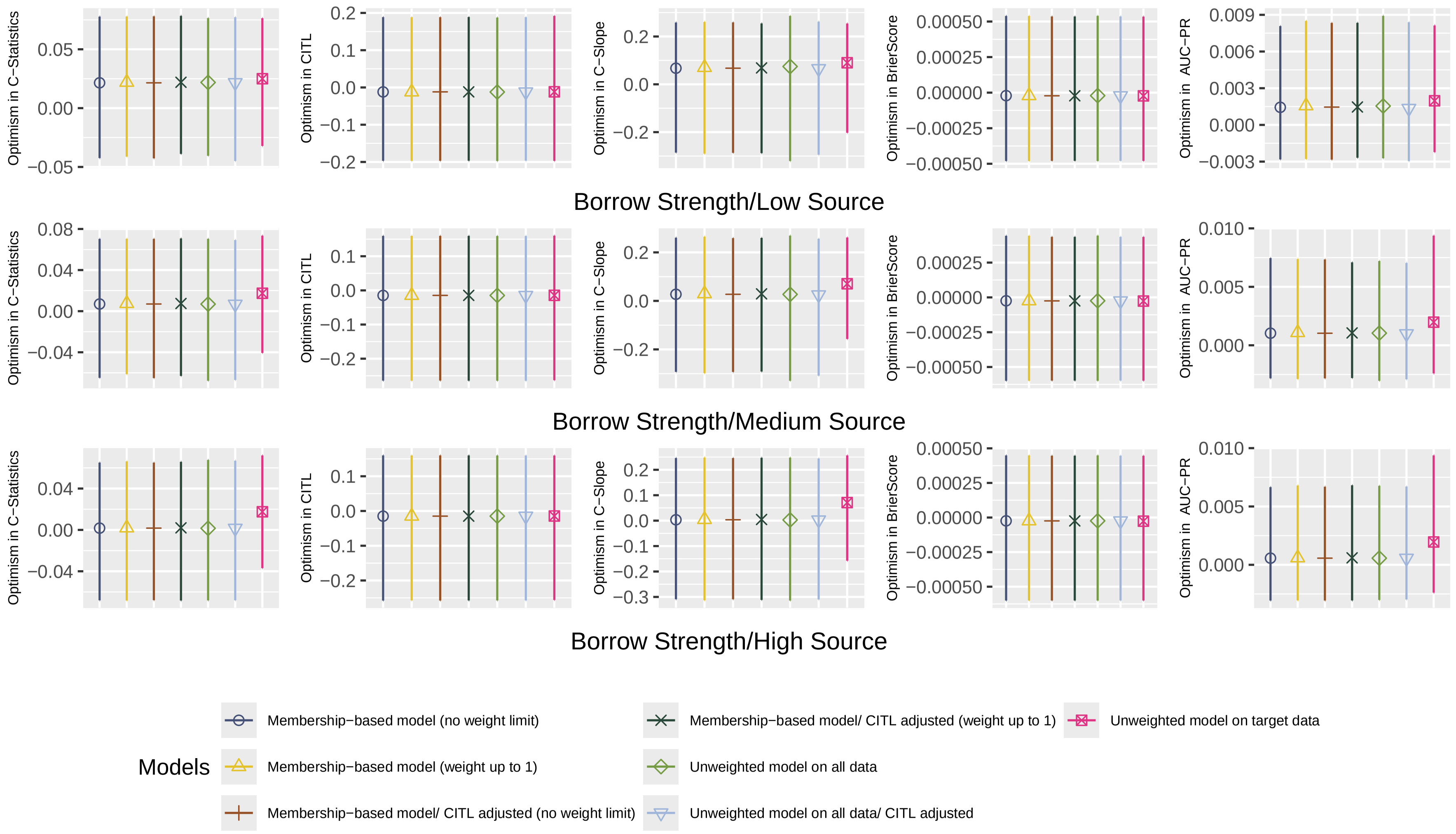

Figure S14 Confidence Intervals of Bootstrap Optimism for Theme 4

7 | BOOTSTRAP PERFORMANCE METRICS

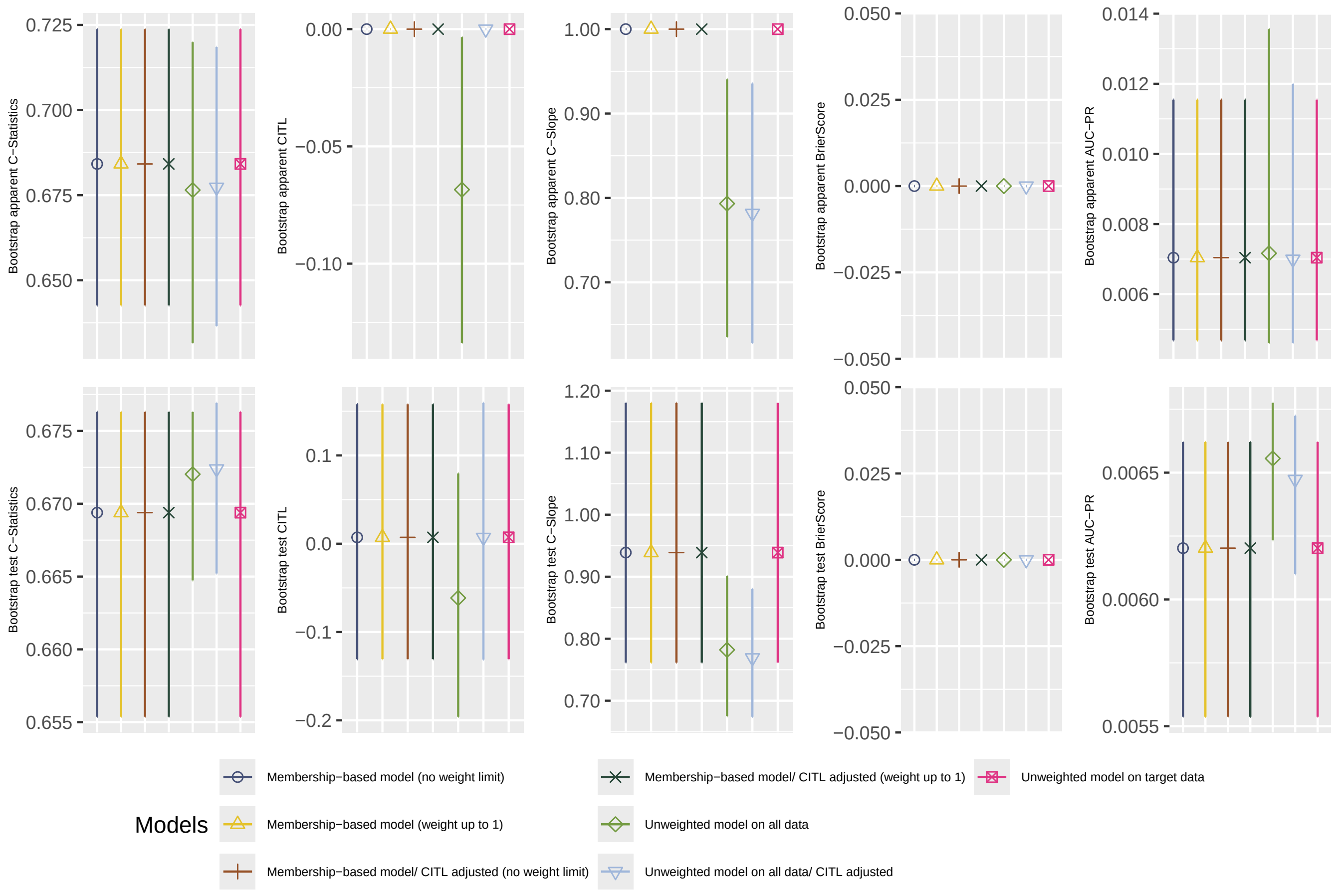

Figure S15 Scenario 1 Bootstrap Performance Metrics' Confidence Intervals

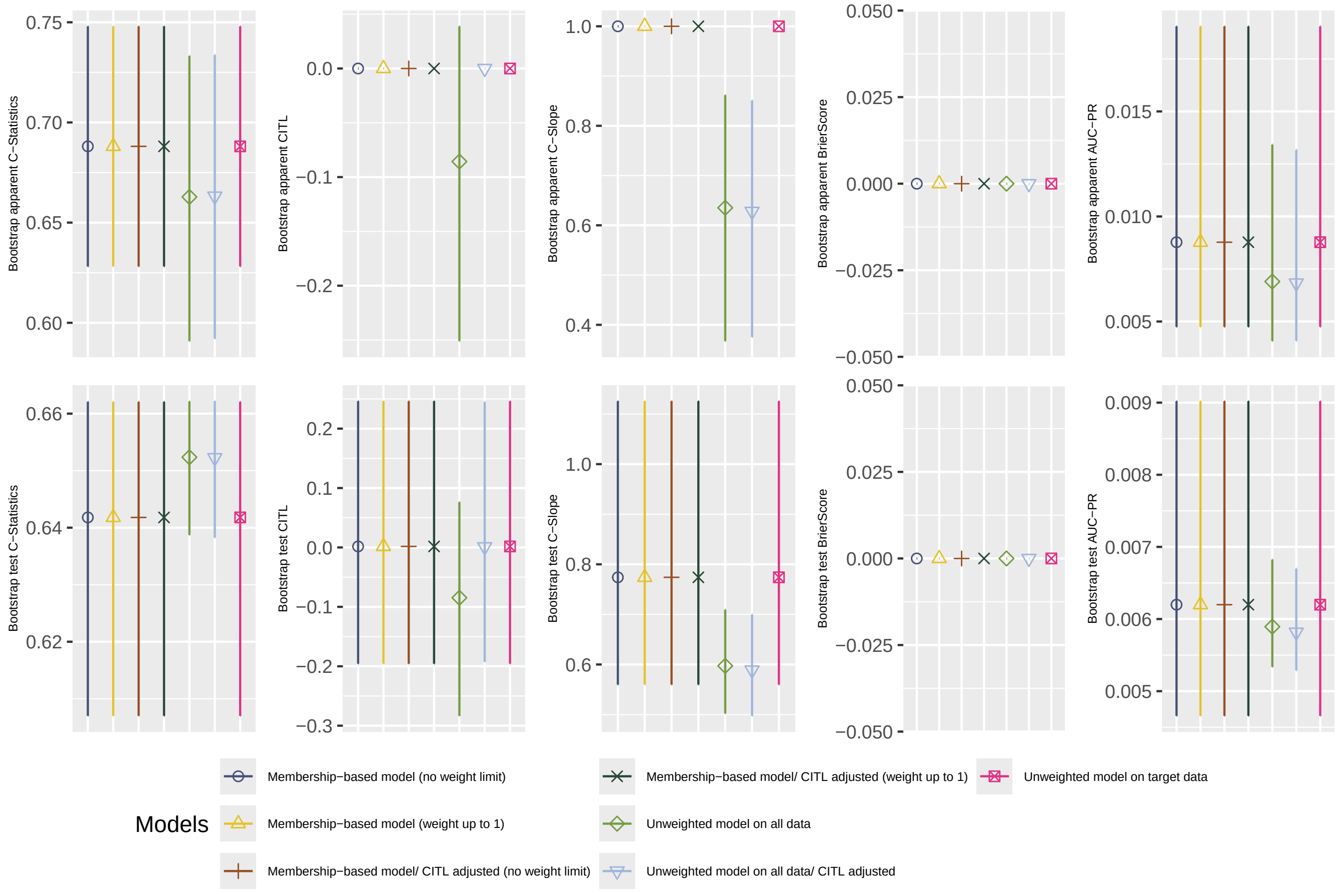

Figure S16 Scenario 2 Bootstrap Performance Metrics' Confidence Intervals

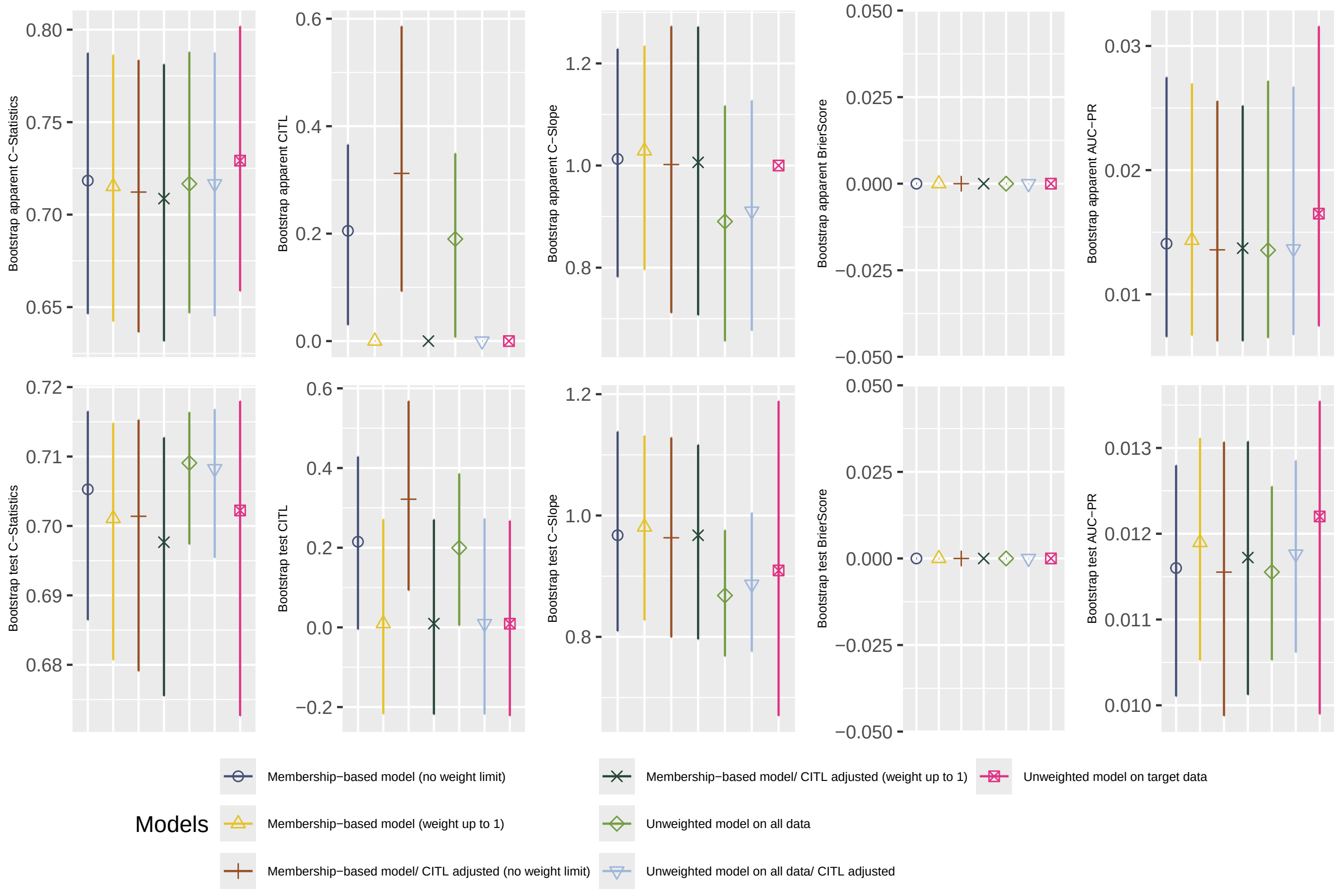

Figure S17 Scenario 3 Bootstrap Performance Metrics' Confidence Intervals

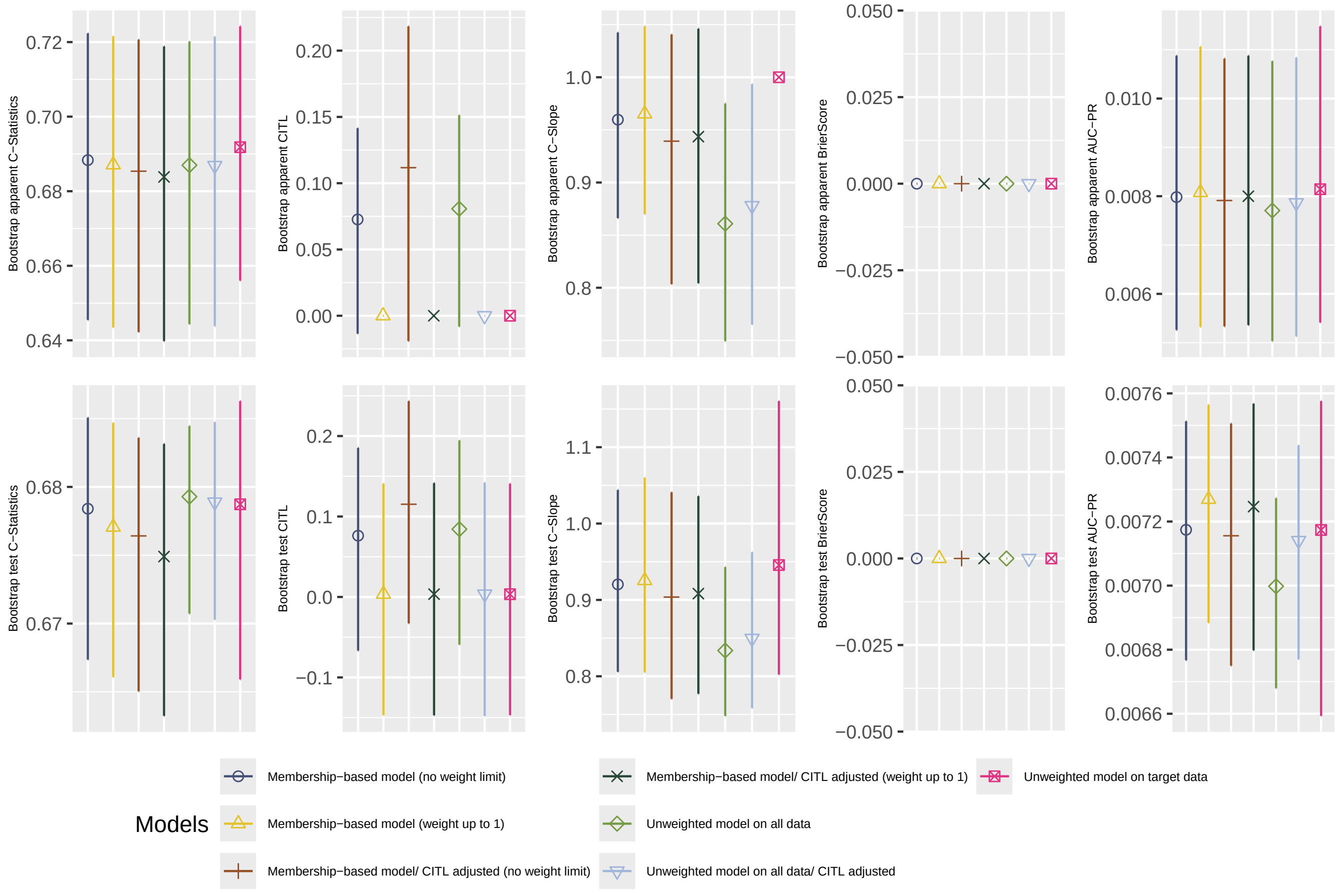

Figure S18 Scenario 4 Bootstrap Performance Metrics' Confidence Intervals

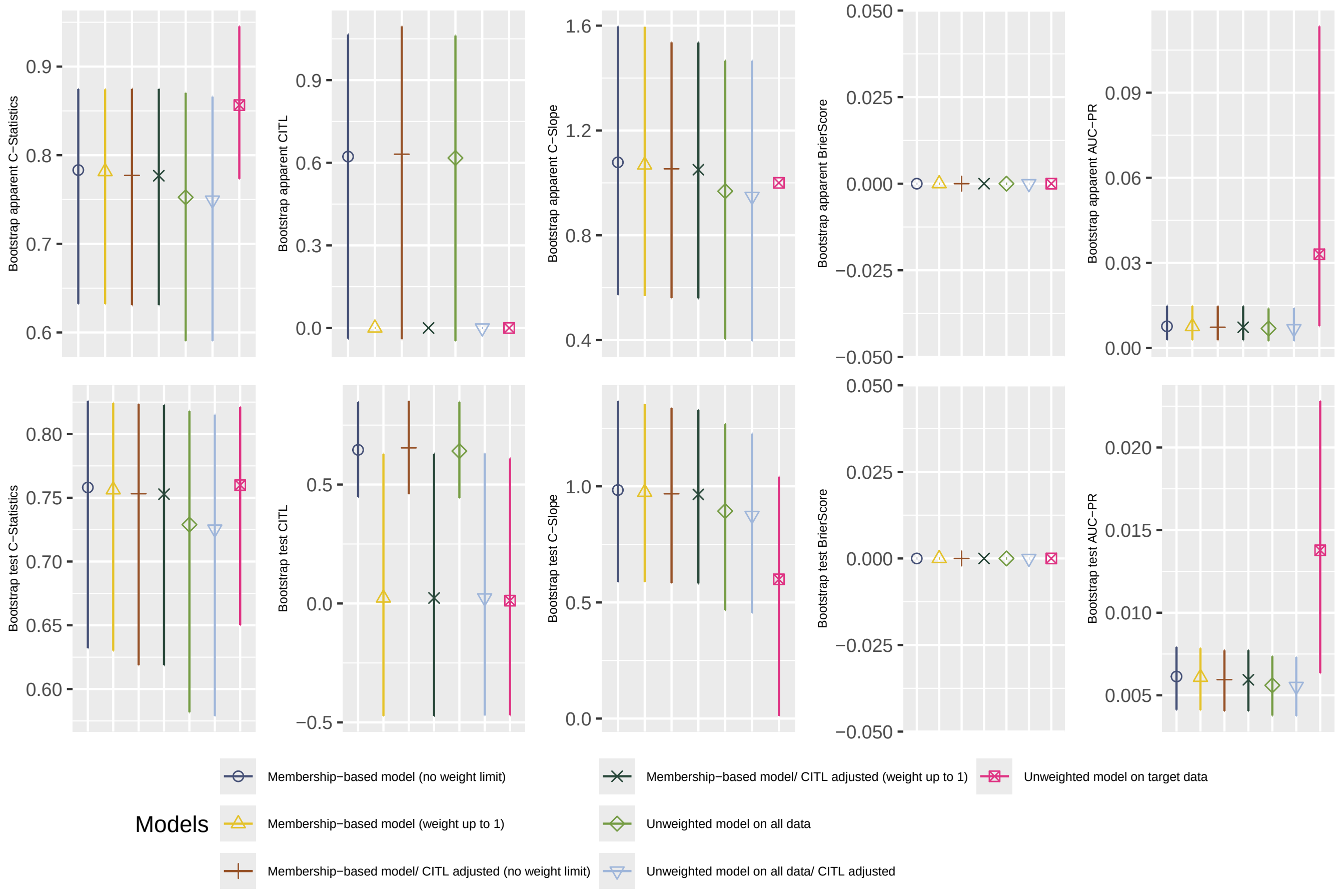

Figure S19 Scenario 5 Bootstrap Performance Metrics' Confidence Intervals

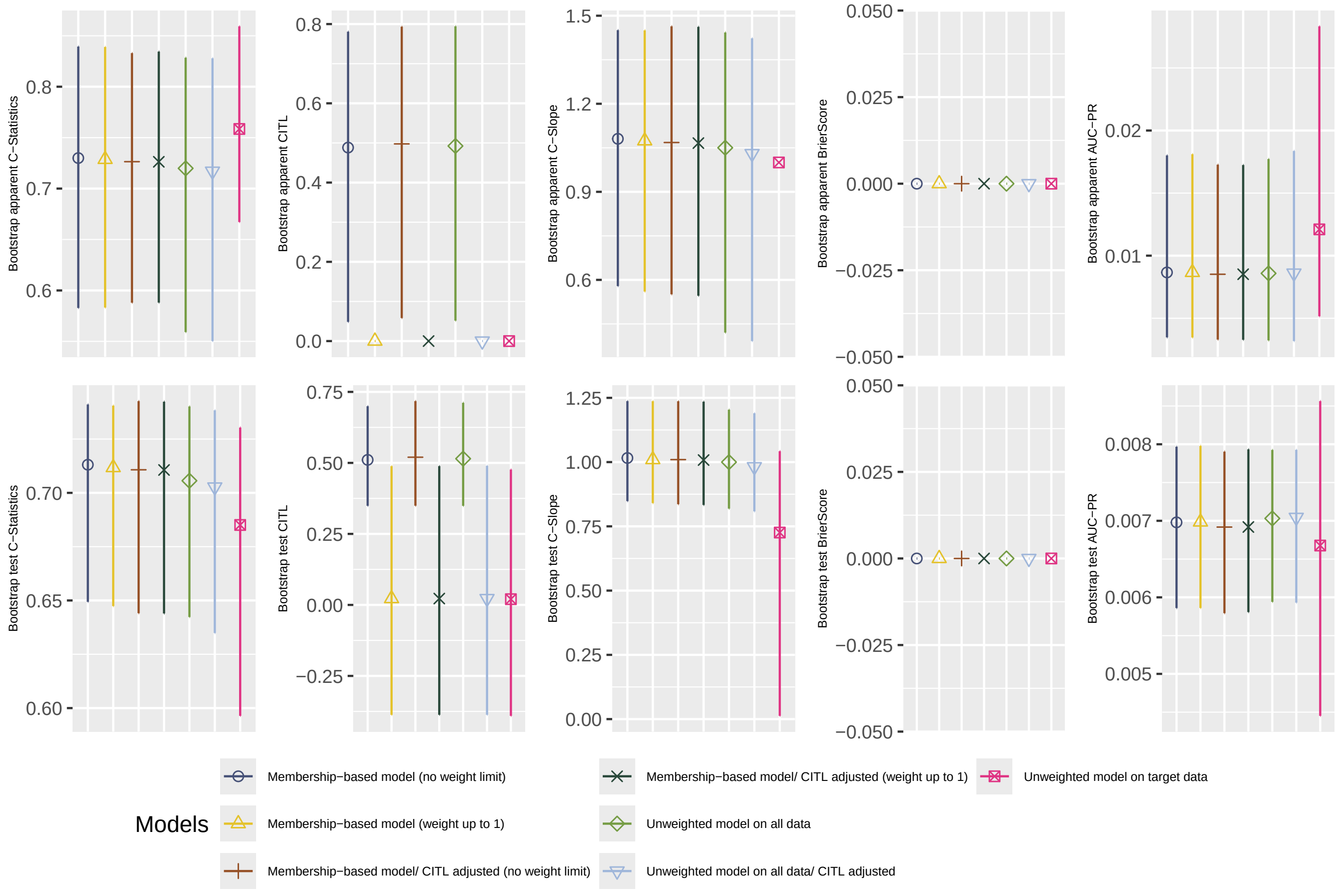

Figure S20 Scenario 6 Bootstrap Performance Metrics' Confidence Intervals

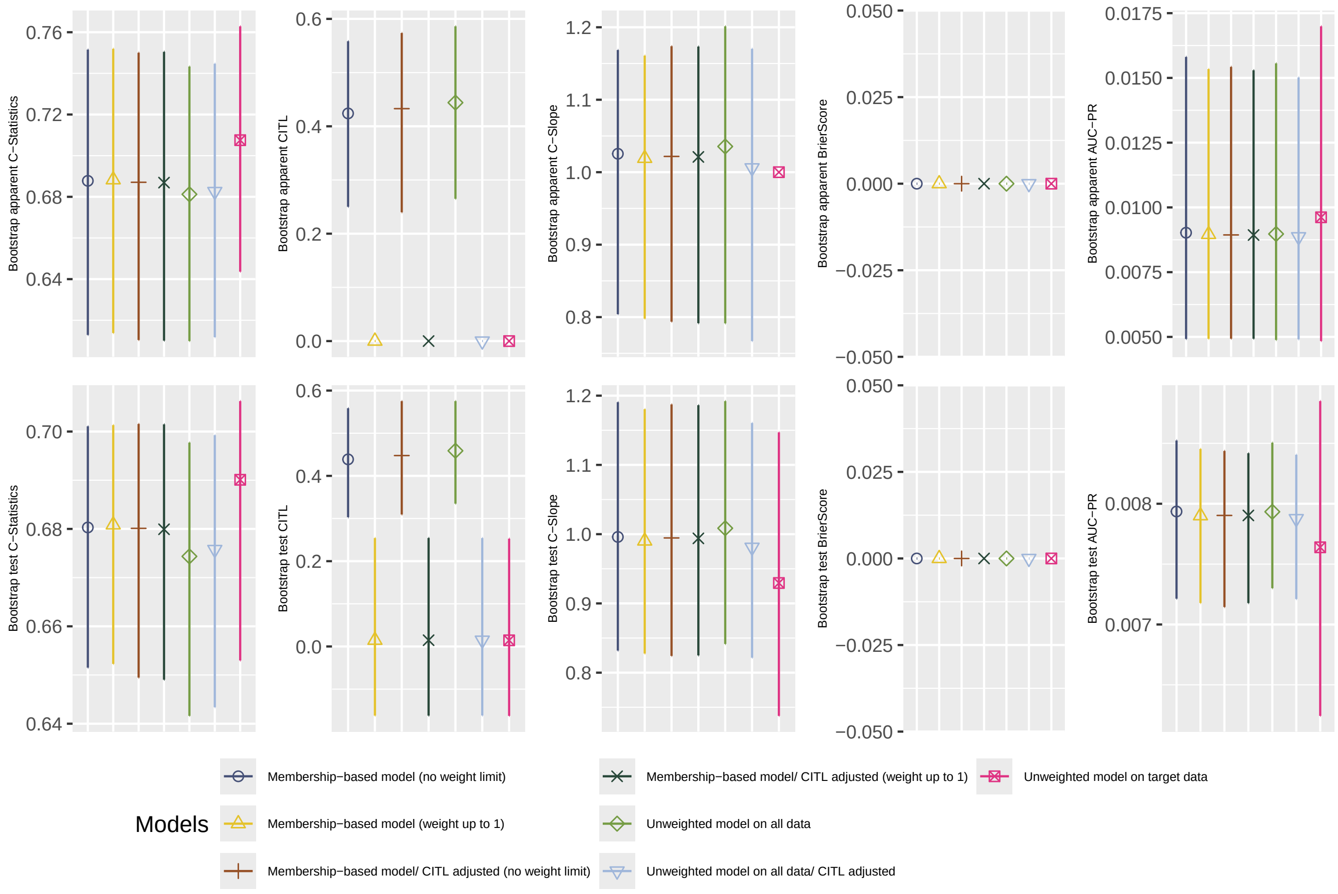

Figure S21 Scenario 7 Bootstrap Performance Metrics' Confidence Intervals

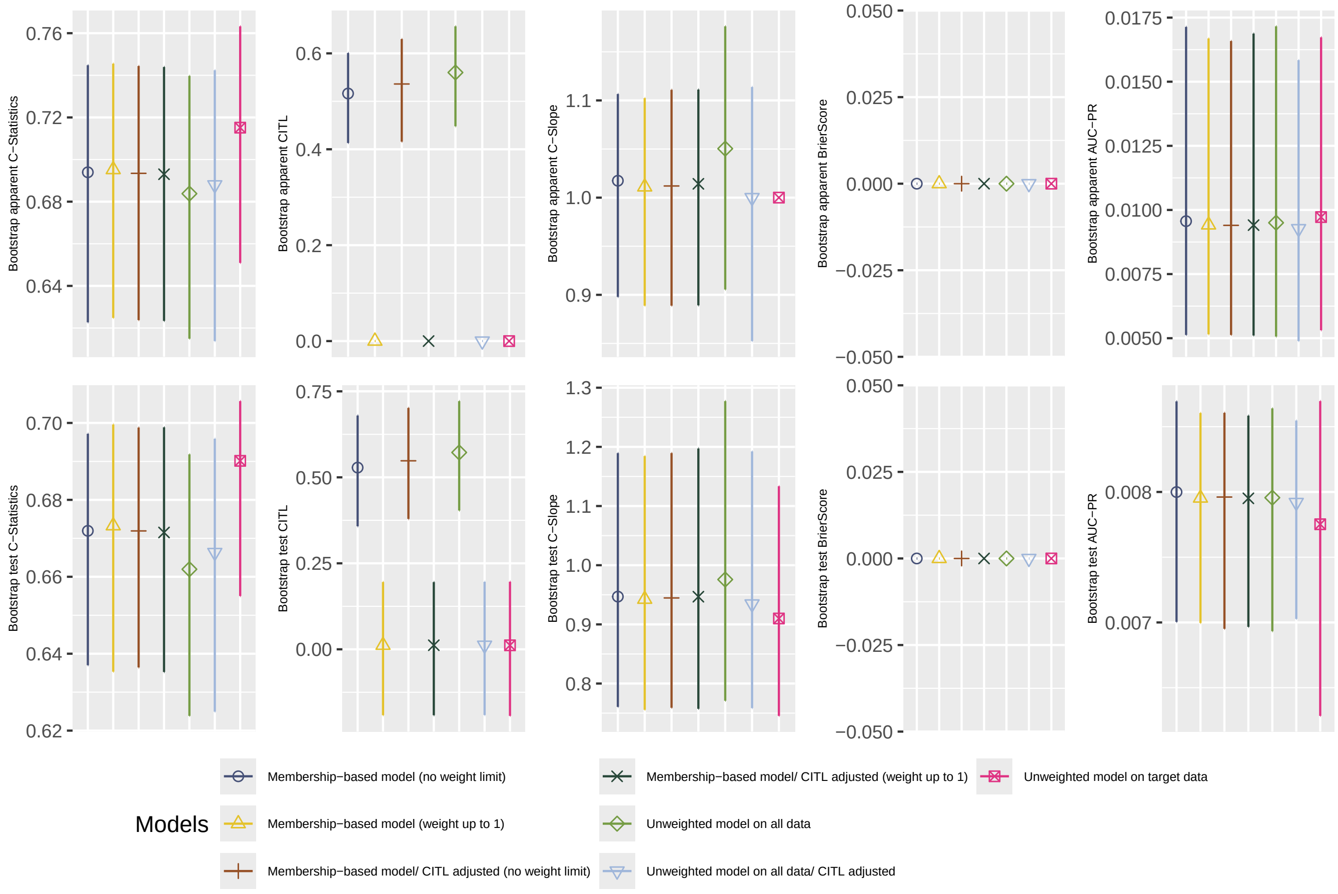

Figure S22 Scenario 8 Bootstrap Performance Metrics' Confidence Intervals

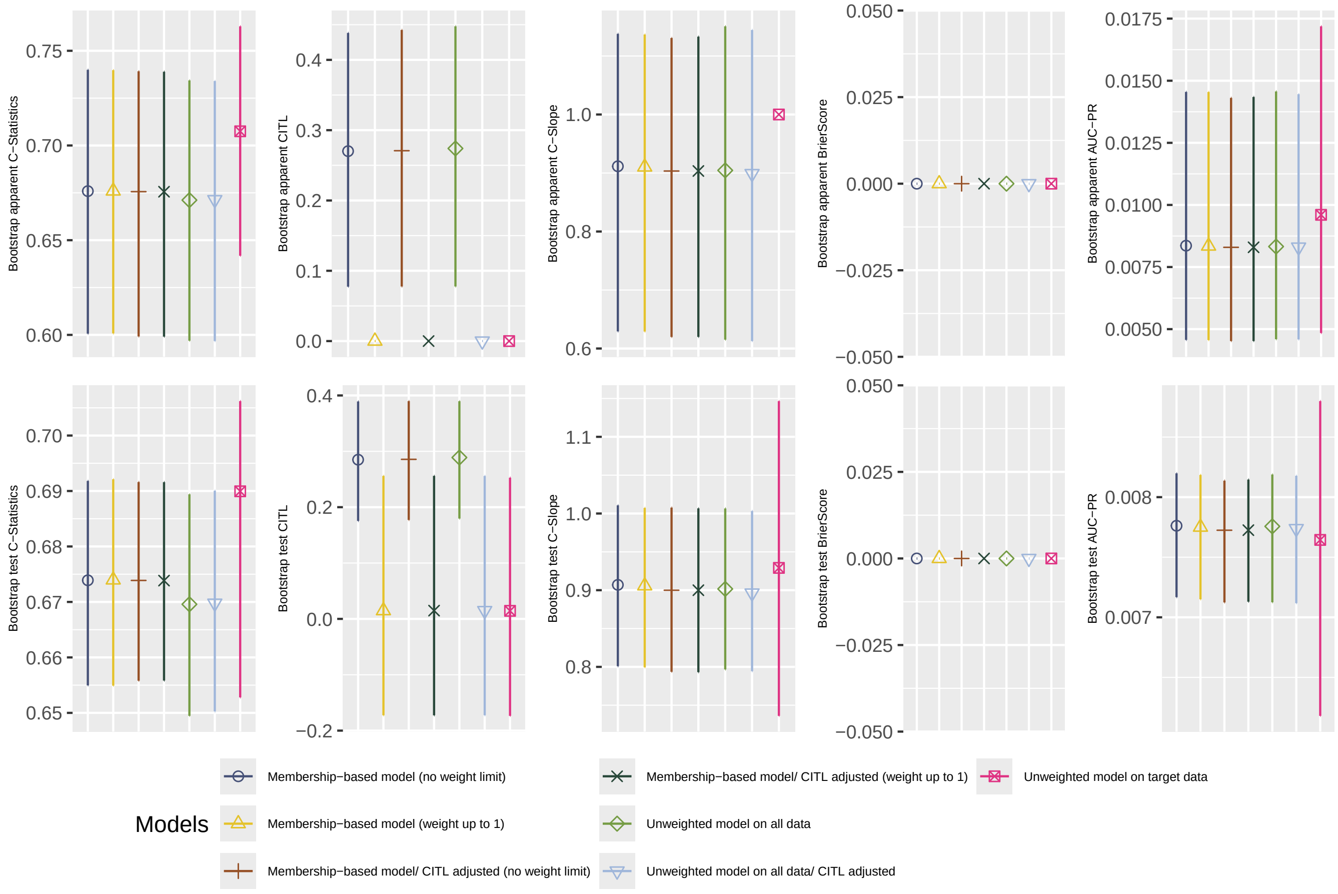

Figure S23 Scenario 9 Bootstrap Performance Metrics' Confidence Intervals

8 | MODELS’ COEFFICIENTS STANDARD ERRORS

Table S3 Scenario 1 Models’ Coefficients Standard Errors

Model	Variables	Std Error (no weight limit)	Std Error (weight up to 1)
Membership-based model	(Intercept)	1.5770	1.5770
	Age	0.0158	0.0158
	Sex	0.1627	0.1627
	AF_atrial_flutter	0.1642	0.1642
	Diabetes	0.1675	0.1675
	BMI	0.0188	0.0188
	LVEF35	0.1719	0.1719
	eGFR	0.0041	0.0041
Membership-based model / CITL adjusted	(Intercept)	2791.4130	2791.4130
	Age	0.0158	0.0158
	Sex	0.1627	0.1627
	AF_atrial_flutter	0.1642	0.1642
	Diabetes	0.1675	0.1675
	BMI	0.0188	0.0188
	LVEF35	0.1719	0.1719
	eGFR	0.0041	0.0041

Table S4 Scenario 2 Models' Coefficients Standard Errors

Model	Variables	Std Error (no weight limit)	Std Error (weight up to 1)
Membership-based model	(Intercept)	2.632	2.632
	Age	0.027	0.027
	Sex	0.272	0.272
	AF_atrial_flutter	0.282	0.282
	Diabetes	0.299	0.299
	BMI	0.032	0.032
	LVEF35	0.306	0.306
	eGFR	0.007	0.007
Membership-based model / CITL adjusted	(Intercept)	1663.105	1663.105
	Age	0.027	0.027
	Sex	0.272	0.272
	AF_atrial_flutter	0.282	0.282
	Diabetes	0.299	0.299
	BMI	0.032	0.032
	LVEF35	0.306	0.306
	eGFR	0.007	0.007

Table S5 Scenario 3 Models' Coefficients Standard Errors

Model	Variables	Std Error (no weight limit)	Std Error (weight up to 1)
Membership-based model	(Intercept)	1.1808	1.2822
	Age	0.0120	0.0124
	Sex	0.1634	0.1900
	AF_atrial_flutter	0.1501	0.1771
	Diabetes	0.1518	0.1772
	BMI	0.0155	0.0184
	LVEF35	0.1600	0.1853
	eGFR	0.0035	0.0041
Membership-based model / CITL adjusted	(Intercept)	1.1500	1.2846
	Age	0.0116	0.0127
	Sex	0.1637	0.1903
	AF_atrial_flutter	0.1505	0.1776
	Diabetes	0.1518	0.1772
	BMI	0.0155	0.0184
	LVEF35	0.1604	0.1857
	eGFR	0.0035	0.0041

Table S6 Scenario 4 Models' Coefficients Standard Errors

Model	Variables	Std Error (no weight limit)	Std Error (weight up to 1)
Membership-based model	(Intercept)	0.8573	0.8953
	Age	0.0079	0.0080
	Sex	0.1251	0.1340
	AF_atrial_flutter	0.1216	0.1320
	Diabetes	0.1193	0.1284
	BMI	0.0126	0.0138
	LVEF35	0.1292	0.1387
	eGFR	0.0028	0.0030
Membership-based model / CITL adjusted	(Intercept)	0.8509	0.8974
	Age	0.0080	0.0083
	Sex	0.1252	0.1341
	AF_atrial_flutter	0.1218	0.1322
	Diabetes	0.1193	0.1284
	BMI	0.0126	0.0138
	LVEF35	0.1294	0.1389
	eGFR	0.0028	0.0030

Table S7 Scenario 5 Models' Coefficients Standard Errors

Model	Variables	Std Error (no weight limit)	Std Error (weight up to 1)
Membership-based model	(Intercept)	1.3336	1.4133
	Age	0.0111	0.0115
	Sex	0.2307	0.2373
	AF_atrial_flutter	0.2518	0.2601
	Diabetes	0.2145	0.2246
	BMI	0.0206	0.0225
	LVEF35	0.2424	0.2455
	eGFR	0.0053	0.0054
Membership-based model / CITL adjusted	(Intercept)	1.3328	1.4092
	Age	0.0111	0.0115
	Sex	0.2308	0.2374
	AF_atrial_flutter	0.2519	0.2602
	Diabetes	0.2145	0.2246
	BMI	0.0206	0.0225
	LVEF35	0.2425	0.2457
	eGFR	0.0052	0.0054

Table S8 Scenario 6 Models' Coefficients Standard Errors

Model	Variables	Std Error (no weight limit)	Std Error (weight up to 1)
Membership-based model	(Intercept)	1.2466	1.3086
	Age	0.0103	0.0107
	Sex	0.2187	0.2239
	AF_atrial_flutter	0.2440	0.2499
	Diabetes	0.2036	0.2112
	BMI	0.0196	0.0210
	LVEF35	0.2301	0.2323
	eGFR	0.0049	0.0050
Membership-based model / CITL adjusted	(Intercept)	1.2463	1.3034
	Age	0.0103	0.0106
	Sex	0.2189	0.2241
	AF_atrial_flutter	0.2440	0.2500
	Diabetes	0.2037	0.2113
	BMI	0.0197	0.0210
	LVEF35	0.2302	0.2326
	eGFR	0.0049	0.0050

Table S9 Scenario 7 Models' Coefficients Standard Errors

Model	Variables	Std Error (no weight limit)	Std Error (weight up to 1)
Membership-based model	(Intercept)	0.98907	1.01964
	Age	0.00824	0.00842
	Sex	0.16659	0.16897
	AF_atrial_flutter	0.18445	0.18655
	Diabetes	0.16525	0.16891
	BMI	0.01610	0.01677
	LVEF35	0.18511	0.18593
	eGFR	0.00384	0.00391
Membership-based model / CITL adjusted	(Intercept)	0.98831	1.01163
	Age	0.00819	0.00835
	Sex	0.16688	0.16927
	AF_atrial_flutter	0.18458	0.18672
	Diabetes	0.16537	0.16900
	BMI	0.01616	0.01679
	LVEF35	0.18539	0.18637
	eGFR	0.00380	0.00386

Table S10 Scenario 8 Models' Coefficients Standard Errors

Model	Variables	Std Error (no weight limit)	Std Error (weight up to 1)
Membership-based model	(Intercept)	1.214	1.244
	Age	0.010	0.010
	Sex	0.204	0.206
	AF_atrial_flutter	0.225	0.226
	Diabetes	0.202	0.205
	BMI	0.020	0.020
	LVEF35	0.226	0.226
	eGFR	0.005	0.005
Membership-based model / CITL adjusted	(Intercept)	1.215	1.229
	Age	0.010	0.010
	Sex	0.204	0.206
	AF_atrial_flutter	0.226	0.227
	Diabetes	0.203	0.206
	BMI	0.020	0.020
	LVEF35	0.227	0.228
	eGFR	0.005	0.005

Table S11 Scenario 9 Models' Coefficients Standard Errors

Model	Variables	Std Error (no weight limit)	Std Error (weight up to 1)
Membership-based model	(Intercept)	0.7424	0.7609
	Age	0.0061	0.0063
	Sex	0.1307	0.1325
	AF_atrial_flutter	0.1311	0.1330
	Diabetes	0.1211	0.1241
	BMI	0.0125	0.0130
	LVEF35	0.1384	0.1392
	eGFR	0.0028	0.0028
Membership-based model / CITL adjusted	(Intercept)	0.7427	0.7596
	Age	0.0061	0.0062
	Sex	0.1308	0.1326
	AF_atrial_flutter	0.1311	0.1330
	Diabetes	0.1211	0.1241
	BMI	0.0125	0.0130
	LVEF35	0.1385	0.1393
	eGFR	0.0028	0.0028
